# Targeting the Ubiquitin System in Glioblastoma

**DOI:** 10.3389/fonc.2020.574011

**Published:** 2020-11-25

**Authors:** Nico Scholz, Kathreena M. Kurian, Florian A. Siebzehnrubl, Julien D. F. Licchesi

**Affiliations:** ^1^ Department of Biology & Biochemistry, University of Bath, Bath, United Kingdom; ^2^ Brain Tumour Research Group, Institute of Clinical Neurosciences, University of Bristol, Bristol, United Kingdom; ^3^ Cardiff University School of Biosciences, European Cancer Stem Cell Research Institute, Cardiff, United Kingdom

**Keywords:** glioblastoma, ubiquitin, E3 ubiquitin ligases, deubiquinating enzymes, PROTAC (proteolysis-targeting chimeric molecule), stem cell, cancer, ubiquitin-proteasome system

## Abstract

Glioblastoma is the most common primary brain tumor in adults with poor overall outcome and 5-year survival of less than 5%. Treatment has not changed much in the last decade or so, with surgical resection and radio/chemotherapy being the main options. Glioblastoma is highly heterogeneous and frequently becomes treatment-resistant due to the ability of glioblastoma cells to adopt stem cell states facilitating tumor recurrence. Therefore, there is an urgent need for novel therapeutic strategies. The ubiquitin system, in particular E3 ubiquitin ligases and deubiquitinating enzymes, have emerged as a promising source of novel drug targets. In addition to conventional small molecule drug discovery approaches aimed at modulating enzyme activity, several new and exciting strategies are also being explored. Among these, PROteolysis TArgeting Chimeras (PROTACs) aim to harness the endogenous protein turnover machinery to direct therapeutically relevant targets, including previously considered “undruggable” ones, for proteasomal degradation. PROTAC and other strategies targeting the ubiquitin proteasome system offer new therapeutic avenues which will expand the drug development toolboxes for glioblastoma. This review will provide a comprehensive overview of E3 ubiquitin ligases and deubiquitinating enzymes in the context of glioblastoma and their involvement in core signaling pathways including EGFR, TGF-β, p53 and stemness-related pathways. Finally, we offer new insights into how these ubiquitin-dependent mechanisms could be exploited therapeutically for glioblastoma.

## Glioblastoma

### Background

Glioblastoma (GBM) is the most common and aggressive malignant primary brain tumor, categorized as grade IV diffuse glioma by the World Health Organization (WHO) (1). Commonly found in the supratentorial region, GBMs constitute 16% of all primary brain tumors and 54% of all gliomas (2). The CBTRUS Statistical Report (2006–2010) estimated the age-adjusted incidence rate of GBM at 3.19/100,000/year in the United States (2) while data from the National Cancer Registration Service and Hospital Episode Statistics for England (2007–2011) estimated the incidence rate at 4.64/100,000/year in England (3). GBM also has a very poor overall survival rate dropping from 28.4% after one year to 3.4% at five years with a median survival of 6.1 months in the English cohort study (3). When stratified by age, median survival was 16.2 months for 20 to 44-year-olds compared to only 3.2 months for 70+-year-olds. Further, incidences are higher in males compared to females with a relative sex ratio of 1.66:1. Overall GBM has a very poor outlook considering that the median age at diagnosis is 64 (2). In 2016, the WHO published its revised classification of tumors of the central nervous system (CNS) which for the first time used histology as well as molecular parameters to guide appropriate tumor classification (1, 4). Here, GBMs are defined as either isocitrate dehydrogenase (*IDH*)-wildtype or *IDH*-mutant, a genotype that in the majority of cases clinically coincides with primary/*de novo* GBM and secondary GBM, respectively (5). Perhaps unsurprisingly, *IDH1* mutations are also very frequent (>80%) in diffuse and anaplastic astrocytomas which are common precursor lesions for recurrent GBM. At the molecular level, IDH mutations result in reduced affinity toward its endogenous substrate, isocitrate, and acquisition of neomorphic enzymatic activity converting α-ketoglutarate into the oncometabolite 2-hydroxyglutarate (6). This gain-of-function has been linked to several oncogenic processes including epigenetic remodeling, which results in the CpG island methylator phenotype (CIMP) (7–9). However, the extent as well as targets of glioma hypermethylation seem to vary considerably when compared to that observed in other IDH^mut^ cancers such as acute myeloid leukemia (AML), possibly explaining why IDH mutational status serves as a favorable prognostic biomarker in GBM only (10). Interestingly, several studies have reported varying methylation patterns between *de novo* and secondary GBM with, for example, promoter methylation of *retinoblastoma protein 1 (RB1)* and *O6-methylguanine methyltransferase (MGMT)* being three-fold and two-fold higher in secondary GBM, respectively (11–15). The epigenetic silencing of the DNA repair enzyme MGMT also serves as IDH-independent prognostic biomarker indicative of increased sensitivity toward temozolomide (TMZ) chemotherapy (16–18). Furthermore, loss of MGMT expression, paired with concomitant TMZ treatment, may select for loss of mismatch repair function resulting in recurrent GBM with hypermutator phenotype (19).

In an attempt to unravel GBM evolution as well as inter- and intra-tumoral heterogeneity, molecular subtyping has been developed as a prognostic strategy. Based on an 840-gene expression profile, GBM samples were grouped into proneural, mesenchymal, classical and neural molecular subtypes (20). Verhaak and colleagues used transcriptomic and genomic profiling to further stratify GBM by identifying patterns of somatic mutations characteristic of individual subtypes. Specifically, *EGFR*, *NF1* and *PDGFRA/IDH* aberrations were found to define classical, mesenchymal and proneural subtypes, respectively. In recent years, single-cell analysis has become well-established and has offered important insights into tumor complexity. Single-cell RNA-sequencing revealed that tumor bulk transcriptomic profiles do not accurately reflect GBM subtypes (20, 21). Transcriptomic profiles characteristic of the four subgroups (i.e. proneural, mesenchymal, classical and neural), differ at the single-cell level within a tumor, providing further support for the heterogeneity of tumors. In agreement with this, data binning of a proneural tumor according to percentage (%) heterogeneity resulted in patient subsets with diverging overall survival. Hence, higher heterogeneity associated with shorter overall survival. Another single-cell RNA-sequencing study found that infiltrating neoplastic cells from the tumor periphery share a common transcriptomic signature despite having distinct dominant subtypes (22). About 1,000 and 250 genes were found down- and up-regulated, respectively, compared to cells from the tumor core, including genes associated with hypoxia (down) or migration/invasion of the interstitial matrix (up). This indicates that despite intratumoral heterogeneity, some mechanisms such as those driving cell invasion are shared between tumors.

### Current Treatments and Future Directions

Treatment of GBM has largely remained unchanged throughout the last decade. A hallmark randomized phase III clinical trial in 2004 by the European Organisation for Research and Treatment of Cancer (EORTC) and the National Cancer Institute of Canada Clinical Trials Group (NCIC), set the following gold standard that is still used today (23, 24). Following maximal safe resection (also referred to as tumor debulking, 84% of patient cohort), patients randomly received radiotherapy alone or radiotherapy with concomitant TMZ chemotherapy followed by six cycles of adjuvant TMZ treatment. The 5-year analysis showed the median survival was 27.2% after two years and 9.8% at five years post treatment commencement. The higher 5-year survival rate compared to previously highlighted epidemiological studies can be explained by the exclusion of 70+-year-olds from the patient cohort. Although surgical debulking followed by radiotherapy and concomitant TMZ chemotherapy remains the current treatment paradigm, several new approaches are being explored including techniques for surgical refinement, immunotherapies and personalized medicine approaches (25–29).

Ongoing efforts in the delineation of the aberrant molecular networks that account for and drive the malignancy and aggressiveness associated with GBM have highlighted key areas that may be exploited therapeutically. In addition to progress made with regards to personalized immunotherapy, the ubiquitin proteasome system has been recognized as one of the most promising fields for novel therapeutics. Proof-of-concept studies have indeed demonstrated that every class of enzymes involved in the ubiquitin-proteasome system can be effectively targeted, including E1-activating, E2-conjugating enzymes, E3 ubiquitin ligases as well as deubiquitinases. With around 1,000 enzymes regulating protein ubiquitination, the number of candidate drug targets is likely to surpass that seen for protein kinases (30). Beyond targeting individual components of the ubiquitin system, new approaches that exploit protein turnover are also being developed and these are bringing new hopes to target the so far “undruggable proteome” (31). In particular, recent developments in proteolysis targeting chimeras (PROTACs) and PROTAC-related molecules such as “molecular glues”, have demonstrated the feasibility of harnessing the endogenous protein turnover machinery for the selective and specific degradation of target proteins. In the next sections, we will discuss key components of the ubiquitin system, in particular E3 ubiquitin ligases and DUBs, in the context of GBM.

## The Ubiquitin System

### Ubiquitin and UBLs

Post-translational modifications serve a plethora of regulatory functions and thus are integral to cellular homeostasis. In particular, protein ubiquitination plays critical roles by regulating protein fate and function. The small protein modifier ubiquitin (76 aa) is highly conserved in eukaryotes, and it is found expressed in all human tissues (32, 33). More recently, analogous systems in the form of or ubiquitin-like proteins (UBLs) were shown to exist in prokaryotes. UBLs are proteins with shared fold homology to ubiquitin, including a globular β-grasp fold, but for which there is little conservation in the primary protein sequence. As many as 10 UBLs, not including paralogs, have been found in humans with the most well-characterized being interferon-stimulated gene (ISG) 15, autophagy-related genes (Atg) 8 and 12, Nedd8 and small-ubiquitin-related modifiers (SUMO) (34, 35). Furthermore, each post-translational modification has its dedicated conjugation and deconjugation machinery, although some overlapping conjugation systems exists between ubiquitin and UBLs (36, 37). For example, ISG15, a 15 kDa interferon-inducible protein modifier, utilizes its own ubiquitin-like modifier-activating enzyme 7 (UBA7 or UBE1L), ubiquitin/ISG15-conjugating enzyme E2 L6 (UBE2L6 or UbcH8) and the E3 ubiquitin ligases HERC5 or TRIM25 (38–42).

Conjugation of ubiquitin or UBLs to substrate proteins is an ancient, highly conserved protein modification. Even though ubiquitin is absent in prokaryotes, at least two families of post-translational modifications, with analogous function but distinct biochemical pathway to ubiquitin, have been described. Pup (prokaryotic ubiquitin-like protein) which mediates the pupylation of substrate lysine residues was identified in the actinobacteria *Mycobacterium tuberculosis/smegmatis* and constitutes the first group (43, 44). The second group includes the archaeal SAMPs (small archaeal modifier proteins) and *Thermus* TtuB (tRNA-two-thiouridine B) which mediate sulfur mobilization (45, 46).

### The Ubiquitin Cascade

The pioneering work of Aaron Ciechanover, Avram Hershko and Irwin Rose led to the discovery of ubiquitination as novel post-translational modification that facilitated subsequent degradation in an ATP-dependent manner (47, 48). Subsequently, the Varshavsky lab made seminal contributions to the fundamental understanding of ubiquitin-mediated protein degradation and the regulation of protein half-life. Specifically, delineation of its biological relevance *in vivo* highlighted ubiquitination as a fundamental requirement for cell viability as well as many cellular pathways including the cell cycle and DNA repair (49, 50).

**Figure 1:**
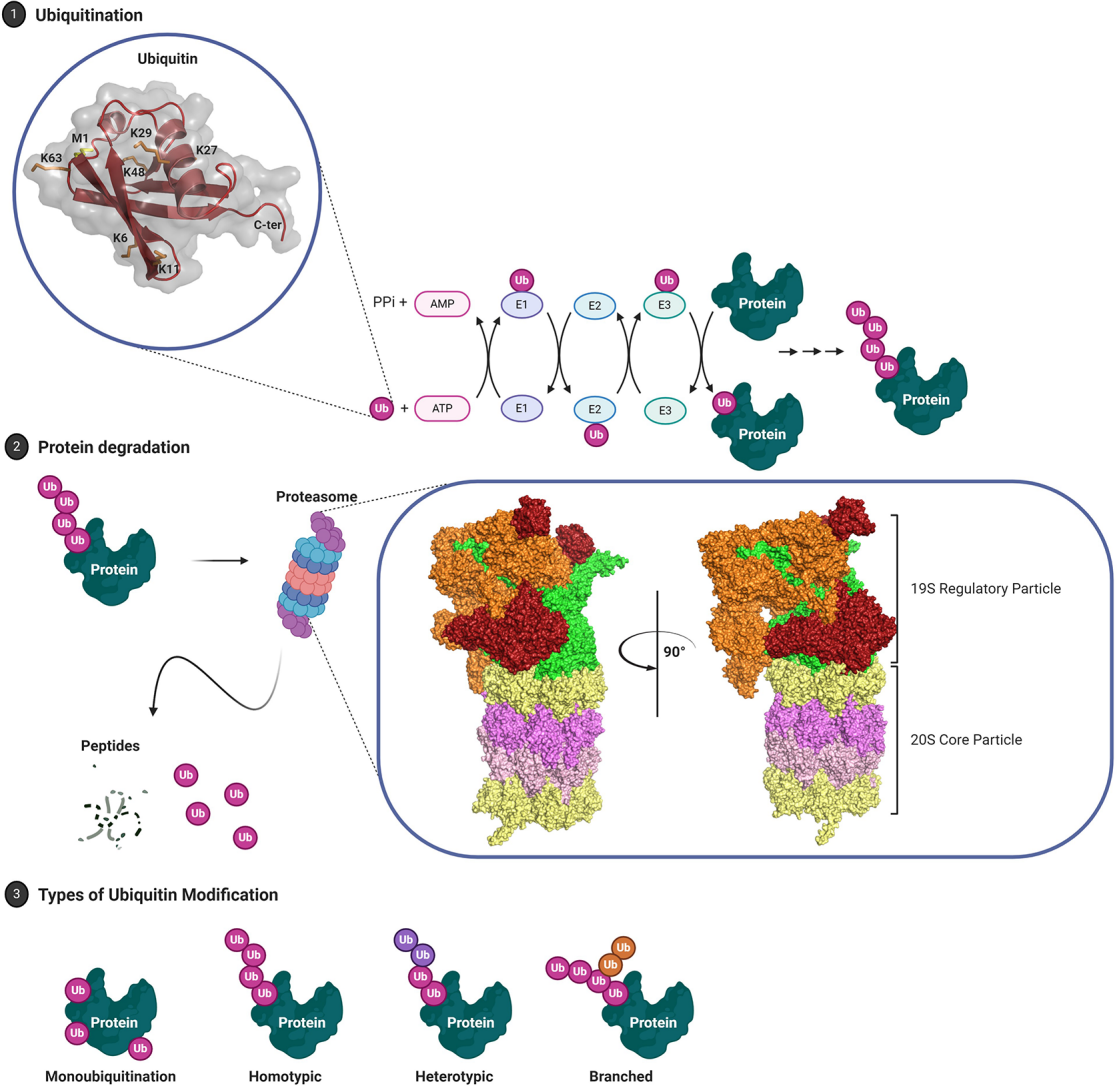
****The Ubiquitin Proteasome System. (1) Post-translational modifier ubiquitin, 8.5 kDa, is thiolated to ubiquitin-activating enzyme (E1) and subsequently transthioesterified to a cognate ubiquitin-conjugating enzyme (E2). E3 ubiquitin ligases either serve as scaffold (RING E3 ligases) or catalytic intermediary (HECT E3 ligases) facilitating covalent linkage of ubiquitin C-terminal Gly^76^ (COOH) to the ϵ-amino group of target lysine residues. (2) Subsequent turnover of ubiquitinated proteins is mediated by the 26S proteasome. Structurally, the proteasome is divided into the 19S regulatory particle, composed of lid and base, and the 20S core particle. Orange: non-ATPase Rpn components (lid); red: ubiquitin receptors Rpn1/10/13 (base); green: AAA+ family ATPases Rpt1–Rpt6 (base); yellow: α heptameric rings, α_1–7_, that constitute gate/substrate entry portal (20S); magenta: β heptameric rings, β_1–7_, that constitute the catalytic chamber. (3) Target proteins may either be monoubiquitinated or modified by chains of varying architecture and composition. The complexity of ubiquitin as a signaling molecule, existing as a single moiety or a complex chains, is matched by an impressive diversity in enzymes including 2 E1s, 40 E2s, ~700 E3s and 99 DUBs encoded by the human genome PDB: 1UBQ, Ubiquitin; 6FVW, 26S proteasome.

Substrate protein modification by ubiquitin is mediated by a hierarchical enzymatic cascade (**Figure 1**). The E1-activating enzyme binds ATP and cofactor Mg^2+^ and catalyzes ubiquitin C-terminal acyl adenylation, which is then transferred to the sulfhydryl group of the catalytic cysteine residue *via* acyl substitution forming a thioester bond (51, 52). E1 ubiquitin loading is complete after a second round of ubiquitin adenylate synthesis forming a ternary complex (53). The kinetically charged thioester-conjugate is then transferred to a catalytic cysteine residue in the ubiquitin conjugation (UBC) domain of a cognate E2 *via* transthioesterification. The thioester bond is susceptible to nucleophilic attack and thus ubiquitin may be transferred to a free substrate lysine (aminolysis) or cysteine residue (transthiolation) (54). Non-canonical transfer may also involve conjugation to serine/threonine residues (oxyester bond) or substrate N-termini (peptide bond) (55–57). The final step of conjugating ubiquitin or UBLs to protein targets is mediated by E3 ubiquitin ligases. This can occur directly by RING E3 ligases which recruit a ubiquitin-loaded E2 and substrate bringing them into close proximity, or indirectly by HECT E3s through an intermediary step where ubiquitin is transthiolated onto a catalytic cysteine residue prior to being transferred to the ϵ-amino group of a substrate lysine residue (58, 59). Single ubiquitin moieties may be attached to a substrate protein at one or multiple sites, monoubiquitination and multi-monoubiquitination respectively, or as ubiquitin chains of varying topology (polyubiquitination), altogether constituting the ubiquitin code (**Figure 1**) (60).

Below, we will summarize our current understanding of the ubiquitin code in terms of the different types of ubiquitin signals that have been identified, their cellular functions, and the “Writers” (E1, E2, E3) and “Erasers” (i.e. DUBs) that regulate this complex but versatile post-translational modification.

## Components of the Ubiquitin System

### E3 Ubiquitin Ligases

#### RING E3 Ligases

**Figure 2:**
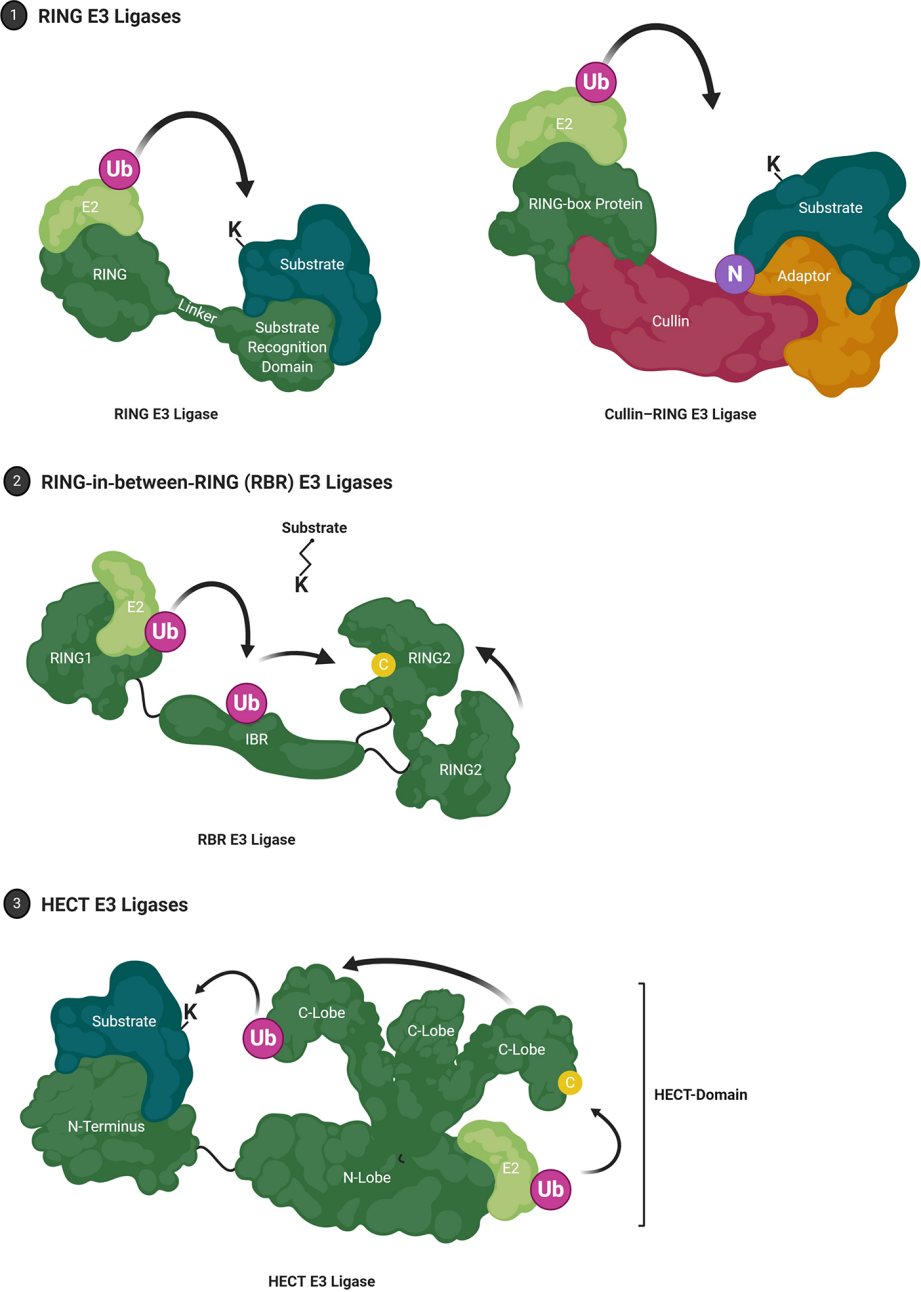
E3 Ubiquitin Ligases. (1) RING (Really Interesting New Gene) E3 ligases facilitate Ub-E2:substrate interaction. RING E3 ligases may also organize into multi-subunit complexes that are commonly composed of Cullins, E2 binding RING-box proteins and an adaptor protein that mediates substrate recognition. Canonically, neddylation (NEDD8) is required to induce the active conformer. (2) RBR (RING-in-between-RING) E3 ligases constitute a hybrid class between RING and HECT E3 ligases where the RING1 domain facilitates E2 interaction while the RING2 domain harbors a catalytic cysteine residue that forms an intermediate thioester. (3) HECT (Homologous to E6-AP Carboxyl Terminus) E3 ligases are characterized by a conserved bi-lobed, catalytic HECT domain. The loaded E2 is bound by the N-lobe where ubiquitin is transferred to the catalytic cysteine residue on the flexible C-lobe. The C-lobe-ubiquitin thioester intermediate rotates toward the substrate which is bound by a substrate-binding domain located N-terminal of the HECT domain.

E3 ubiquitin ligases catalyze the final step of ubiquitination and impart substrate specificity through protein family diversity. Over 700 E3 ubiquitin ligases are encoded by the human genome and based on their mode of action these have been grouped in four major families: Really Interesting New Gene (RING), Homologous to E6-AP Carboxyl Terminus (HECT), RING-in-between-RING (RBR), and U-box E3 ubiquitin ligases (**Figure 2**).

RING E3s are the largest family with >600 members and are characterized by their zinc finger RING domain (**Figure 2**) (61). The conserved crossbraced structure of the RING domain is facilitated by two Zn^2+^ ions and may adopt monomeric or (homo/hetero) dimeric conformations (62–64). Generally, RING domains transiently interact with the E2 UBC domain *via* a shallow groove constituted by the central α-helix and two adjacent loop regions, thus competing with E1-E2 interaction while other domains are responsible for substrate recruitment (62, 65). Instead of strictly acting as an E2-substrate scaffolding protein, RING E3 ligases also have a passive catalytic capacity and have been shown to facilitate the proximity required for isopeptide bond formation through manifold dynamic conformational rearrangements (66–69). For example, the E2 Ubc13 (Ube2N) and its cofactor Mms2 do not require an E3 to coordinate substrate ubiquitination but exhibit increased reaction kinetics and differential substrate specificity in the presence of E3 ligase TRAF6 (63, 70). A major fraction of RING E3 ligases, the Cullin-RING E3 ligase superfamily, organize into large, multi-subunit proteins (**Figure 2**) such as the SCF (Skp1, Cullin 1, F-box protein) complex or anaphase-promoting complex/cyclosome (APC/C) [reviewed in (71)].

#### U-Box E3 Ligases

U-box E3s have a comparable mechanism to RING E3s, utilizing a structurally similar domain they can also operate as monomers or function as subunits of multimeric protein complexes. In contrast to RINGs, U-box E3s lack the conserved cysteine and histidine Zn^2+^-chelators at the RING domain-binding interface and instead utilize a hydrophobic binding groove constituted by hydrogen bonding networks and polar amino acids (72, 73). A well-researched example is carboxy-terminus of Hsc70 interacting protein (CHIP), a co-chaperone that acts as quality control for misfolded proteins by ubiquitinating Hsp70 and Hsp90 associated substrates (74, 75). The N-terminal tetratricopeptide repeat (TPR) domain mediates Hsp70/Hsp90 interaction, while a coiled-coil domain facilitates CHIP dimerization allowing the homodimer U-box domain to interact with its cognate E2 (76, 77).

#### RBR E3 Ligases

RING-in-Between RING (RBR) E3 ligases are a comparably small family with the human genome encoding about 12 such proteins [reviewed in (78, 79)] Originally thought to employ a similar mechanism to canonical RING ubiquitination, RBR E3 ligases were rather shown to be hybrids of RING and HECT E3 ligases (80). The RING1 domain mediates interaction with a ubiquitin-loaded E2, while the RING2 domain forms a thioester intermediate *via* a catalytic cysteine residue (**Figure 2**). The two-step mechanism is reminiscent of HECT-mediated ubiquitination, although the domain structure differs. Several RBRs have been shown to function in this manner, including HHARI, HOIP and Parkin (81–83). Additionally, Parkin which contributes to neurodegeneration in Parkinson’s disease, but also GBM, can carry out E2-independent monoubiquitination and remains active in the absence of its RING1 domain (84–86). Nevertheless, all RBRs are thought to share stringent intramolecular auto-inhibitory mechanisms. In the case of Parkin, an N-terminal ubiquitin-like domain induces a closed, auto-inhibited conformation by binding to the IBR-RING2 linker region (87). Parkin substrates have been shown to interact with the ubiquitin-like domain, indicating substrate-induced activation (88, 89).

#### HECT E3 Ligases

HECT E3 ligases can be divided into 16 subfamilies with a total of 28 members encoded by the human genome (90). The HECT family of E3 ligases were discovered during the investigation of E3 ligase E6AP (UBE3A), which then became its first member (91–93). Here, the human papillomavirus (HPV) virulence protein E6 was shown to hijack the mammalian E3 ligase, altering its substrate specificity toward tumor suppressor p53 as well as other regulatory proteins (94, 95). HECT-mediated ubiquitination requires an intermediate step, whereby ubiquitin is first transferred onto the E3 catalytic cysteine residue *via* a thioester bond, prior to conjugation onto protein substrates. This allows HECT-type E3 ligases to veto any linkage preference conferred by E2 conjugating enzymes (96). The approximately 40 kDa bi-lobed HECT domain is composed of an N-lobe and a C-lobe, with the N- and C-lobes being separated by a hinge glycine residue (**Figure 2**) (97). While the C-lobe contains the catalytic cysteine residue, the larger N-lobe primarily mediates E2 interaction as evidenced by the crystal structure of E6AP in complex with UBCH7 (97). In the context of at least some HECT E3s such as NEDD4, the N-lobe can also provide a binding interface for ubiquitin itself and this might promote processivity during ubiquitin chain extension (98). These and other structural studies have emphasized that conformational flexibility of the HECT domain is key to bringing the catalytic cysteine of the E3 in close proximity with that of the E2 and thus to enable ubiquitin transfer (97, 99, 100).

Even though HECT E3 ligases share the highly conserved HECT domain, they display considerable diversity in their N-terminal domains which are thought to play important roles in substrate targeting as well as E3 ubiquitin ligase regulation (101). This has been most well-characterized for the NEDD4 HECT E3 ligase family (NEDD4, NEDD4.2, ITCH, SMURF1, SMURF2, WWP1, WWP2, NEDL1 (HECW1) and NEDDL2 (HECW2)) which contain an N-terminal Ca^2+^-dependent/independent lipid-binding domain (C2 domain) and between two to four WW domains in addition to the C-terminal HECT domain (102, 103). Type I WW domains within NEDD4 family proteins bind a multitude of substrates by engaging PY motifs (PPxY) as well as other proline-rich motifs (104–106). C2 domains mediate targeting of the E3 to the phospholipid bilayer but may also confer substrate specificity (107, 108). Various inter- and intramolecular interactions are prominent in the regulation of HECT E3 ligase activity (109). For example, C2-HECT domain interaction within SMURF2 results in the canonical closed/autoinhibitory conformation that may be outcompeted if a substrate is available (110). Further, NEDD4 forms an autoinhibitory trimer *via* a conserved α1-helix domain, which is contrasted by E6AP trimerization that constitutes its active conformer (111, 112).

### Deubiquitinases

In many aspects, the cellular counterpart to E3 ubiquitin ligases, DUBs remove ubiquitin moieties from substrate proteins ensuring reversibility of the post-translational modification. Around 100 DUBs have been identified in eukaryotes and are divided into seven evolutionary conserved families (USP, JAMM/MPN, OTU, MJD/Josephin, UCH, MINDY, and ZUP1) (113). DUBs are predominantly thiol proteases with a catalytic cysteine residue or in the case of the JAMM family metalloproteases coordinate a Zn^2+^ ion in the active site (114). DUBs may display substrate specificity and/or ubiquitin linkage specificity. The reversibility of protein ubiquitination was first demonstrated in 1982 by the observation that histone H2A is deubiquitinated during mitosis and re-ubiquitinated during the G1 phase (115). Later, the first DUB, YUH1, was identified in *S. cerevisiae* and the lack of obvious phenotypic changes suggested the existence of additional DUBs (116). DUBs have since been implicated in most if not all cellular processes including DNA repair, signal transduction and innate immunity (113).

DUBs also play a crucial role in the *de novo* synthesis of ubiquitin and thus maintenance of the cellular ubiquitin pool. Human ubiquitin is encoded by four genes expressing the ubiquitin precursors UBB, UBC, UBA52 and UBA80. UBB and UBC exist as head-to-tail linked ubiquitin polymers with a C-terminal extension, while UBA52 and UBA80 are ubiquitin monomers fused to the ribosomal proteins L40 and S27A, respectively (117–119). Processing of ubiquitin precursors is carried out by multiple DUBs and likely serves as additional quality control checkpoint. Ribosomal fusion precursors are post-translationally cleaved by UCHL3, USP7 and USP9X, while ubiquitin multimer precursors are processed by USP5 and OTULIN (120).

In addition, DUBs also carry out another important “housekeeping” function by recycling ubiquitin as part of the UPS and the endocytic pathway (121, 122). Upon recognition of ubiquitinated cargoes by ubiquitin receptors on the proteasome lid, including Rpn10, Rpn13 and Rpn1, the polyubiquitin signal is cleaved off by proteasomal DUBs including UCH37 (UCHL5), Usp14 and PSMD14 (Rpn11) (**Figure 1**) (123). As demonstrated for PSMD14, catalytic activity is in direct competition with ubiquitin unfolding by the proteasomal AAA-ATPases (124). This results in mechanochemical coupling of the two processes, where substrate translocation accelerates conformational switching of PSMD14 into its active β-hairpin conformer. Mechanistically, PSMD14 exists as a dimer with the pseudo-DUB PSMD7 which is subject to steric inhibition by the 20S entry port (125). Therefore, even though PSMD14 may not exert linkage specificity *in vitro*, it may only catalyze *en bloc* chain removal, at least in the context of the proteasome (126). The recycling of ubiquitin on the 19S cap is part of a highly orchestrated series of events which leads to cargo unfolding by AAA-ATPases and translocation into the 20S core particle where proteolysis takes place (127).

In other cellular contexts, DUB linkage specificity is of more importance. For example, NFκB signaling relies on the K63-linked polyubiquitination of adaptor proteins for the recruitment of the TAB–TAK1 kinase complex, M1-linked polyubiquitination of NEMO by the linear ubiquitin chain assembly complex (LUBAC), and the K48-linked polyubiquitination of IκBα, the key effector kinase mediating activation of the pathway (128). To regulate NFκB activity, ubiquitin chain disassembly is orchestrated by OTU DUBs OTULIN and CYLD which exert M1 and M1/K63 specificity, respectively (129, 130). Importantly, loss of function of OTULIN drives inflammation and autoimmunity in mice and leads to OTULIN‐related autoinflammatory syndrome (ORAS) in humans (131). Similarly, deficiency in CYLD or A20, a master regulator of NFκB, lead to overt pathway activation and inflammation (132).

## The Ubiquitin Code

Ubiquitin can be conjugated to a substrate lysine residue *via* its C-terminal glycine residue but may also be conjugated to itself. Polyubiquitin chains can thus be assembled through any of its lysine residues (K6, K11, K27, K29, K33, K48, and K63) as well as the N-terminal methionine residue (M1 or linear chains) (**Figure 1**). Although ubiquitin smears were observed in the initial study by Hershko and colleagues, it took further efforts to confirm the existence of polyubiquitin chains (48). These were first identified as K48-linked polyubiquitin chains attached to lysine residues on short-lived proteins which targeted them for proteolytic degradation by the 26S proteasome in an ATP-dependent manner (133–136). Importantly, each of these linkages has now been identified in yeast and mammalian cells by mass spectrometry (137, 138). Over the last two decades, atypical chains (assembled through linkages other than K48 or K63), as well as more complex polyubiquitin signals such as heterotypic and branched chains, have also been reported, emphasizing the complexity and diversity of ubiquitin as a signaling molecule (60, 139, 140).

Homotypic K48-linked ubiquitin chains canonically signal for proteasomal degradation and also represent the most abundant linkage-type (135, 137, 141). Additional linkage-types that mediate proteasomal targeting include K29, which may also drive lysosomal degradation, and perhaps surprisingly, monoubiquitination and K63 which have been predominantly associated with non-proteasomal functions including endocytosis and autophagy (141–144). Indeed, K63-polyubiquitin chains have been primarily implicated in protein complex assembly which includes TRAF6, Ubc13-Uev1A and TRIKA2 (TAK1, TAB1 and TAB2) that associates with IκB kinase (IKK). Here, K63-polyubiquitination stimulates phosphorylation of IKK by TAD1 which leads to the K48-linked polyubiquitination and degradation of IKK and transcriptional activation of NFκB gene targets (145, 146). Other K63 signaling functions include but are not limited to regulation of DNA repair (also K27) (147–149), protein sorting (150, 151) and mRNA splicing (152) and translation (153). Similarly, K48 chains can also mediate non-proteasomal functions as evidenced by the stabilization of the yeast transcription factor M4 by K48-polyubiquitination (154).

Heterotypic ubiquitin chains may either present with “mixed” or “branched” topology. For example, the E3 ligase complex LUBAC, which assembles linear M1-linked ubiquitin chains, also forms K63/M1-linked hybrid ubiquitin chains. In the context of innate immunity, these hybrid chains mediate activation of the canonical IKK complex, but have also been shown to play a role in the TNFR1 and NOD1 signaling networks through modification of RIP1 and RIP2 kinases, respectively (128, 155). Meyer and Rape showed that APC/C assembles K11/K48-branched ubiquitin chains through its E2s Ube2S and Ube2C resulting in a degradation signal that is superior to homotypic K11/K48 chains (156). To achieve polyubiquitin chains of branched topology, the cooperation of enzymes with differing linkage specificity is key. This is the case with ITCH and Ubr5 which assemble K63-linked chains and K48-linked chains, respectively, resulting in K48/K63-branched ubiquitination of proapoptotic regulator TXNIP (157). Similarly, ubiquitin branching has also been demonstrated in yeast where the E4 enzyme Ufd2p catalyzes K48-linked multi-monoubiquitination of Ufd4p-assembled K29-linked polyubiquitin chains as part of the ubiquitin fusion degradation (UFD) pathway (158, 159). In eukaryotes, K29/K48 branched chains have so far been demonstrated to play a role in targeting substrates to the UPS and ERAD (160, 161). Interestingly, the UPS appears well-equipped for processing these more complex chains, with a recent study showing that the proteasome-associated DUB UCH37 is a debranching DUB with important roles during proteasomal degradation (162). Through continued advancements in mass spectrometry-based techniques including the quantification of polyubiquitin linkage composition (e.g. ubiquitin-AQUA, Middle-Down MS), and the dissection of polyubiquitin architecture (e.g. UBICREST, Ub-Clipping, TUBE, TR-TUBE), new insights into the structure and function of branched ubiquitin chains are now possible (138, 163–167).

The versatility of ubiquitin as a signaling molecule makes it a prime target for cancer cells that seek to escape physiological regulation. Indeed, deregulation of the ubiquitin system is often observed in tumor-suppressing pathways (e.g. overactivation/expression of an E3 ligase leading to the ubiquitin-dependent degradation of a protein with tumor suppressive function) as well as tumor-promoting pathways (e.g. inactivation of an E3 ligase leading to the stabilization of oncoproteins). Thus, E3 ubiquitin ligases and DUBs in particular have emerged as therapeutic candidates, offering the possibility to more accurately control the activity of a given pathway in contrast to targeting protein degradation as a whole through proteasomal inhibition. The contribution of ubiquitin signaling to GBM tumorigenesis is currently not well understood. In the next section, we will highlight ubiquitin-dependent mechanisms relevant to GBM and discuss these in the context of EGFR, TGF-β, p53 and stemness-related pathways. **Table 1** and **Table 2** provide a comprehensive overview of E3s and DUBs implicated in GBM, respectively.

**Table 1:** E3 ubiquitin ligases in Glioblastoma (GBM).

Name	Function	Target/Substrate	Reference
**RING E3 Ligases**			
A20	Inhibition of TRAIL-induced apoptosis *via* RIP1 K63-polyubiquitination	RIP1	(168)
APC/C	Cell cycle regulation Regulation of GSCs *via* CDC20-APC/SOX2 signaling axis	CDC20, KIF11, SOX2	(169–171)
BIRC3	Hypoxic adaptation in mesenchymal GBM	?	(172)
BRE1	Polyubiquitination of tumor suppressor p42 Ebp1	Ebp1	(173)
c-Cbl	Regulation of αPix-mediated cell migration and invasion Negative regulation of PI3K-AKT pathway *via* neddylation of c-Src	αPix, c-Src	(174, 175)
CHIP	Regulation of PI3K/AKT signaling *via* the CHIP/miR-92b/PTEN regulatory network CSN6-CHIP-EGFR signaling axis	EGFR, PTEN	(176, 177)
CUL4B	Knockdown induced G1 arrest and decreased expression of cyclin D1	?	(178)
HOIL-1L	Hypoxic adaptation *via* HIF1-dependent PKCζ degradation	PKCζ	(179)
IAP1	Regulation of c-Myc and NFκB signaling	ASK1, IAP2, MAD1/4, TRAF2	(180)
IAP2	Negatively regulates XIAP stabilization of mature Smac and Bcl10 Regulation of NFκB signaling	XIAP	(181)
LZTR1/CUL3	Regulation of the RAS/MAPK signaling cascade	RAS	(182)
MDM2/HDM2	Overexpression provides escape from p53-regulated growth control Associated with multi-drug resistant phenotype Regulation of HIF1α in a PTEN-PI3K-AKT-dependent manner	HIF1α, p53	(183–185)
MEX3A	Regulates tumor suppressor RIG-I	RIG-I	(186)
NRDP1	Negative regulator of non-canonical Wnt signaling	Disheveled, Vangl1/2	(187)
nXIAP	Regulation of NFκB activation and apoptosis Inhibits IAP2 autoubiquitination	Caspase 3/7/9, IAP2, TAK1/TAB1	(181, 188)
PRAJA1	Overexpressed in gliomas with inverse relationship to cell cycle regulator and apoptotic genes Mediates degradation of CIC, possibly contributing to hyperactive RTK/Ras/ERK signaling	Capicua (CIC)	(189, 190)
PRAJA2	Degradation of NDR/LATS kinase component Mob, attenuating the Hippo cascade and sustaining tumor growth	Mob	(191)
RAD18	Knockdown reduced cell viability and invasive capacity	?	(192)
RBX1/ROC1 (SCF)	Silencing induces G2-M arrest, apoptosis and senescence	?	(193)
RNF123	miR-155-5p-RNF123-NF-κB1-p50-SerpinE1 signaling axis	NFκB?	(194)
RNF135	Knockdown induced G0/G1 arrest and attenuation of p-ERK activation	RIG1	(195)
RNF138	Downregulation attenuated tumour growth and reversed EMT, possibly *via* Erk signaling pathway Degradation of rpS3 provides mechanism for radioresistance	rpS3	(196, 197)
RNF144	Epigenetic regulation Downregulation under hypoxic stress in mesenchymal GSCs increases cell survival	BMI1	(198)
RNF168	Reduced expression of RNF168 in MTAB-deficient GBM cells leads to H2AX destabilisation	H2AX	(199)
SCF^β-TrCP^	Regulation of GBM stem cell maintenance/differentiation Nuclear mislocalization induces PI3K/Akt and Wnt/β-catenin pathway dysregulation	β-catenin, PHLPP1, REST	(200–202)
SCF^Fbw7^	Tumor suppressor commonly mutated in GBM Enhances BNIP3-mediated hypoxic cell death *via* Mcl-1 degradation Silencing reduced G2/M arrest and apoptosis	Aurora-A/B, c-Jun, c-Myc, Cyclin E, Mcl-1, Notch1/4, SOX9	(203–206)
SCF^FBXL14^	Antagonizes USP13-mediated c-Myc stabilization, negatively regulating GSC self-renewal	c-Myc	(207)
SCF^FBXO16^	Low expression in GBM results in active Wnt signalling	β-catenin	(208)
SCF^SKP2^	Regulation of p27 stability *via* PTEN/PI3-kinase pathway Senescence and cell cycle regulation Knockdown resulted in chemosensitization and reduced sphere formation ability	p21^Cip1/Waf1/Sdi1^, p27^KIP1^	(209–212)
SHPRH	Tumor suppressive phenotype	PCNA	(213)
SIAH1	Proapoptotic role in GBM p53^WT^ cells	HIPK2, p27	(214, 215)
TRAF2	Silencing induces G2-M arrest and radiosensitization NO induced CREB phosphorylation *via* IRE1-α/TRAF2/JNK axis Regulation of NFκB signaling	IRE1-α, SGEF/Rac1	(216–218)
TRIM3	Regulation of stem cell dynamics and asymmetric cell division Regulation of c-Myc and Musashi–Notch signaling	?	(219)
TRIM8	Regulation of stemness *via* STAT3 signaling	PIAS3	(220, 221)
TRIM9s	Enhances p38 signaling *via* K63-uniquitination of MKK6	MKK6	(222)
TRIM11	Overexpression promoted a stem-like phenotype Exerts oncogenic effect through EGFR pathway	?	(223)
TRIM14	Promotes EMT by regulating ZEB2 stability	ZEB2	(224)
TRIM33	Degradation of nuclear β-catenin	β-catenin	(225)
TRIM45	Stabilizes p53 *via* K63-polyubiquitination	p53	(226)
VHL	Regulation of JAK/STAT and hypoxic signaling and Wnt/β-catenin pathway Regulation of angiogenesis *via* VEGF	β-catenin, HIF-1α	(227–229)
**RING-Between-RING E3 Ligases**			
PARKIN	PARK2 mutations lead to cyclin E dysregulation and mitotic instability Degradation of APE1 under cellular stress Negative regulation of EMT *via* ZEB1	APE1, Cyclin E	(85, 230, 231)
**HECT E3 Ligases**			
HECTD1	Negative regulation of Wnt pathway	Adenomatous polyposis coli?	(232)
HERC3	Promotes autophagy-induced EMT *via* SMAD7/TGF-β signaling	SMAD7	(233)
HUWE1 (Mule)	Regulation of N-Myc transcriptional activity	N-Myc	(234, 235)
ITCH/AIP4	Regulation of FLIPs stability *via* PTEN-Akt-AIP4 pathway	FLIPs	(236)
NEDD4	FoxM1B-induced degradation/downregulation of PTEN	PTEN	(237)
SMURF1	Knockdown reduced cell invasion Correlates with poor prognosis	?	(238)
SMURF2	Dysregulation of TGF-β signaling	TβR-I	(239)
UBE3B	Knockdown sensitized cells to chemotherapeutic and resulted in mitochondrial fragmentation Regulation of mitochondrial oxidative stress response	Calmodulin	(240, 241)
UBE3C	Knockdown decreased cell migration and invasion Ubiquitination of tumor suppressor ANXA7	Annexin A7	(242)
**SUMOylase**			
NUSAP1	Stabilizes DNA damage sensor ATR Increased chemotherapeutic resistance	ATR	(243)
PIAS1	Mediates STI1 nuclear retention during DNA-damage response Regulates stability of RNA helicase, DDX39B	DDX39B, STI1	(244, 245)

Name function, target/substrates and references are depicted for E3 ubiquitin ligases implicated by the literature to play a role in GBM.

**Table 2:** Deubiquitinases in Glioblastoma (GBM).

Name	Function	Target/Substrate	Reference
**USPs (Ubiquitin Specific Proteases)**			
USP1	Promotes stem cell maintenance *via* stabilization of ID1 and CHEK1 β-catenin–USP1-EZH2 axis links aberrant β-catenin signaling with EZH2-mediated gene epigenetic silencing	CHEK1, EZH2, ID1, ID2	(246–248)
USP2a	Stabilizes MDM4, which regulates p53 activity	MDM4	(249)
USP3	Regulation of EMT and invasion *via* stabilization of Snail Promotes radioresistance *via* Smo-USP3-Claspin axis	Claspin, Snail,	(250, 251)
USP4	Negatively regulates p53 stability Knockdown downregulates PCNA, Bcl-2, upregulates Bax Activates ERK pathway	?	(252, 253)
USP5	In GBM, USP5 generates a shorter isoform 2 that promotes growth and migration	?	(254)
USP7 (HAUSP)	Prevents neuronal differentiation in NPCs by stabilizing REST Promotes tumorigenesis *via* LSD1	LSD1, REST	(255, 256)
USP8 (hUBPy)	Regulates FLIPs stability and TRAIL sensitivity *via* Akt-USP8-AIP4 axis Identified as GSC fitness gene	AIP4	(257, 258)
USP9X	Regulates survival by stabilizing Mcl-1 Knockdown reduces levels of Bcl-2 family members, XIAP and Survivin Maintains mesenchymal identity by stabilizing ALDH1A3	ALDH1A3, Mcl-1, SOX2	(259–262)
USP10	Overexpressed and correlates with poor survival	?	(263)
USP11	EGFR-vIII epigenetically silences USP11, a negative regulator of cell cycle, *via* PI3K/AKT-HDAC1/2 axis Stabilizes tumor suppressor PML	PML	(264, 265)
USP13	Maintenance of GSCs by stabilizing c-Myc	c-Myc	(207)
USP15	Binds Smurf2 and stabilizes TβR-I Regulates WNT pathway Knockdown downregulates mesenchymal markers and proliferative/invasive capacity	HECTD1, Smurf2, TβR-I	(232, 239, 266)
USP18	Negative regulator of IFN response; possibly promotes apoptotic resistance	?	(267)
USP22	GSK3β and USP22-dependent KDM1A stabilization is required for the demethylation of histone H3K4, thereby repression of BMP2, CDKN1A, and GATA6	KDM1A	(268)
USP28	Promotes tumorigenesis by stabilizing c-Myc	c-Myc	(269)
USP48	Sonic Hedgehog pathway-USP48-Gli1 loop promotes tumorigenesis	Gli1	(270)
CYLD	Regulates hypoxia-mediated inflammation Stabilizes RIPK1 for cell survival	RIPK1	(271, 272)
**OTUs (Ovarian Tumor Family)**			
A20 (TNFAIP3)	Regulator of cell survival and the NF-κB pathway Overexpressed in GSCs	?	(273)
**JAMM/MPN+ Family**			
BRCC3	Knockdown reduces growth, migration and TMZ resistance	?	(274)
CSN6	Promotes proliferation and metastasis by stabilizing EGFR Promotes CHIP auto-ubiquitination	CHIP, EGFR	(176)

Name function, target/substrates and references are depicted for E3 ubiquitin ligases implicated by the literature to play a role in GBM.

## The Ubiquitin System in Glioblastoma

### Epidermal Growth Factor Receptor

EGFR amplification and mutations rendering the receptor constitutively active are commonly observed in GBM. Most common are deletions of exons 2-7 (EGFRvIII^Δ6-273^), which result in constitutive activation of receptor signaling as well as global epigenomic and transcriptomic remodeling with chromatin landscape analysis revealing that activation of 2245 putative enhancers was specific to EGFRvIII (275). Also, EGFR amplification (44%) and point mutations that target the extracellular domain (R108K, A289V/D/T and G598D; 24%) are frequently observed (TCGA, PanCancer Atlas). Likewise, loss of the negative Akt regulator PTEN is associated with poor survival (276). Interestingly, in addition to mutations causing loss of expression or enzymatic activity, L320S and T277A have been found to dysregulate PTEN stability and cellular localization by altering the membrane-binding regulatory interface resulting in increased polyubiquitination (277).

Indeed, EGFR stability and downstream signaling are subject to the ubiquitin regulatory network (**Tables 1**, **2**). In GBM, the DUB CSN6, a subunit of the COP9 signalosome complex (CSN), mediates EGFR stabilization and was also shown to be overexpressed in GBM tumor samples (176). CSN6 may also destabilize EGFR-interacting E3 ligase CHIP by promoting its autoubiquitination (278). Interestingly, in a non-GBM context, it is well-established that the multi-subunit metalloprotease CSN regulates the neddylation of CRLs (279). Here, its CSN6 subunit has been associated with the degradation of tumor suppressor proteins including c-Myc and p53 (280, 281). Another E3 ligase, TRIM11, also regulates EGFR levels and TRIM11 expression correlated closely with glioblastoma stem cell (GSC) markers Nestin and CD133 and promoted tumorsphere formation (223).

### TGF-β Signaling

**Figure 3:**
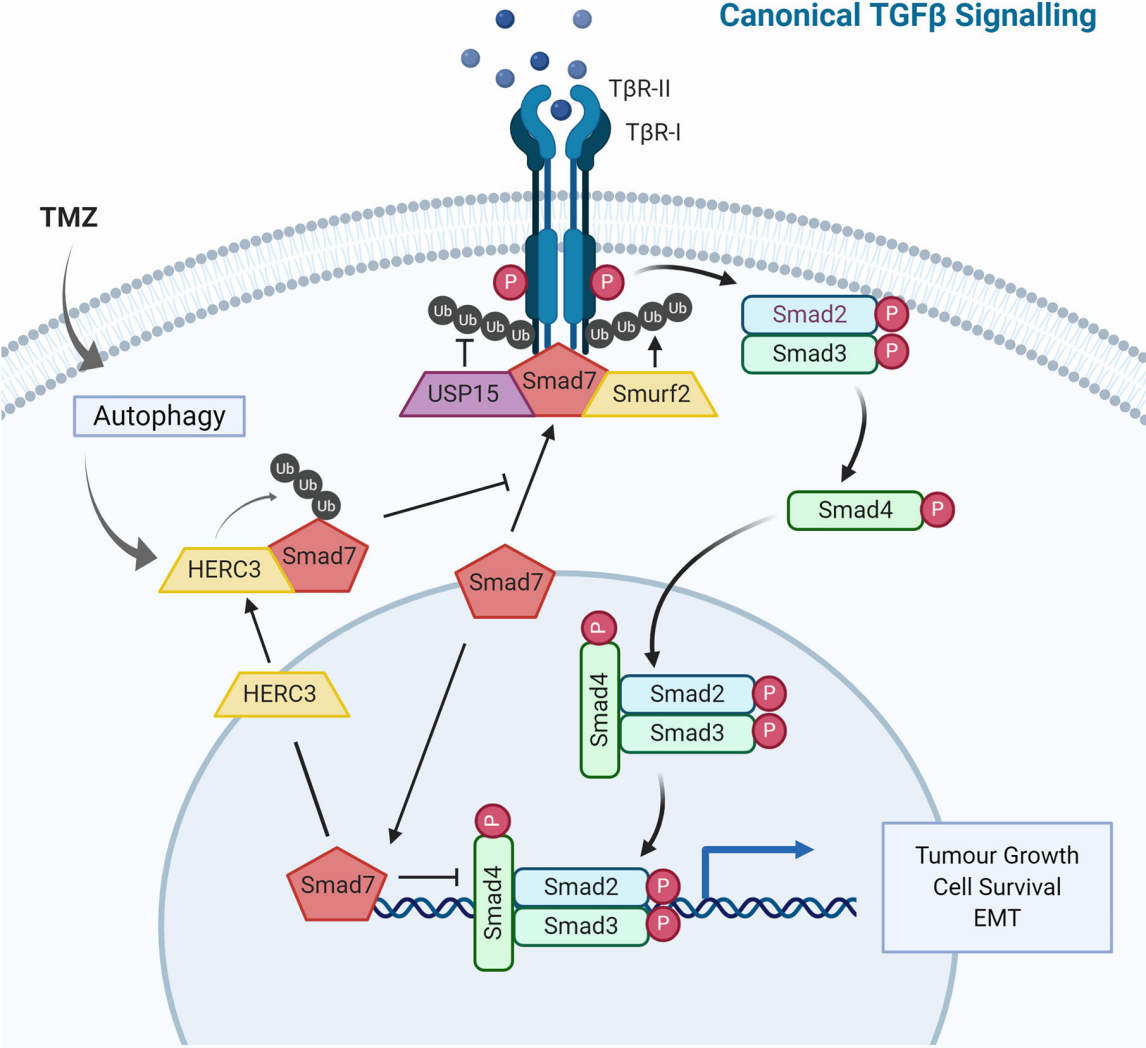
TGF-β Signaling and Ubiquitin in Glioblastoma. The TGF-β signaling cascade is tightly regulated by the ubiquitin-proteasome system. Illustrated are E3 ubiquitin ligases and deubiquitinases that not only regulate TGF-β signaling under physiological conditions but have also been shown to contribute to dysregulation observed in glioblastoma.

Aberrant rewiring of tumor-suppressing TGF-β signaling that induces potent cell cycle arrest to one that promotes cell growth and EMT is characteristic of tumor progression. It has been shown that in patients with high-grade gliomas TGF-β signaling is highly active and this is associated with poor prognosis (282). The canonical TGF-β pathway signals through receptor-regulated Smads (R-Smads), but the receptor may also directly cross-communicate with non-canonical pathways including MAPK, PI3K or RHO-like GTPases (283). TGF-β signaling is subject to tight regulation by ubiquitination (**Figure 3**). The inhibitory Smad protein Smad7 functions as a negative feedback loop by complexing with TβR-I and blocking R-Smad phosphorylation, or by binding to the promoter region of *PAI1*, blocking functional SMAD2/3-DNA complex formation. Further, Smad7 also serves as a docking site for the HECT E3 ligase Smurf2 and DUB USP15 (239, 284). This E3-DUB pair is yet another example of signaling regulation by ubiquitination and a quick and responsive mechanism to regulate pathway activity/output (285). Smurf2 suppresses TGF-β signaling by targeting TβR-I for proteasomal degradation, while USP15 opposes TβR-I polyubiquitination thus stabilizing the receptor complex. Indeed, USP15 knockdown decreased tumorigenic potential in GBM, while more than 2.5 copies of USP15 conferred significantly poorer life expectancy in patients (239).

The physical and functional interaction between Smad7 and the HECT E3 ligase HERC3 has been shown to play a role in chemoresistance observed in GBM. Concomitant TMZ chemotherapy is a standard treatment for high-grade gliomas and has been shown to induce autophagy-mediated cell death (286). Nonetheless, in a subset of tumor cells, this catabolic process may also have pro-survival effects rendering the tumor chemoresistant (287). HERC3 has also been shown to play a key role in autophagy-induced EMT, a core molecular mechanism for drug resistance in GBM (233, 288). Experiments in GBM cells showed that TMZ-induced autophagy resulted in significant up-regulation of TGF-β signaling and subsequent expression of mesenchymal markers. Specifically, autophagy upregulated HERC3 expression which resulted in HERC3-mediated Smad7 K63-polyubiquitination and subsequent autolysosomal degradation. HERC3 binds Smad7 *via* its RCC4–7 domains (aa156–366) and in addition to targeting cytoplasmic Smad7, HERC3 also disrupted the inhibitory interaction of nuclear Smad7 with the promoter region of *PAI1* (289).

The HECT E3 ligase Smurf1 carries out very similar functions to Smurf2 by also binding to Smad7, however it does not co-precipitate with USP15 indicating a contextually different, USP15/Smurf2-independent role (239, 290). Downstream of receptor complex activation, the DUB USP10 drives TGF-β signaling by stabilizing Smad4 which has been linked to increased metastatic potential in hepatocellular carcinoma (291). Another HECT E3 ligase, NEDD4L, recognizes the phosphorylated PPXY motif of Smad2/3 *via* its WW domain, resulting in polyubiquitin-mediated turnover and reduced TGF-β signaling output (292). This mechanism is specific to the canonical TGF-β pathway since it requires the phosphorylation of p-Smad2/3 by TGF-β-activated CDK8/9, which does not regulate non-canonical TGF-β pathways. These and additional ubiquitin-dependent mechanisms have been implicated in TGF-β signaling, and their dysregulation is frequently observed in GBM as well as other cancers, therefore opening new avenues for therapeutic intervention (293).

### p53 Regulation

**Figure 4:**
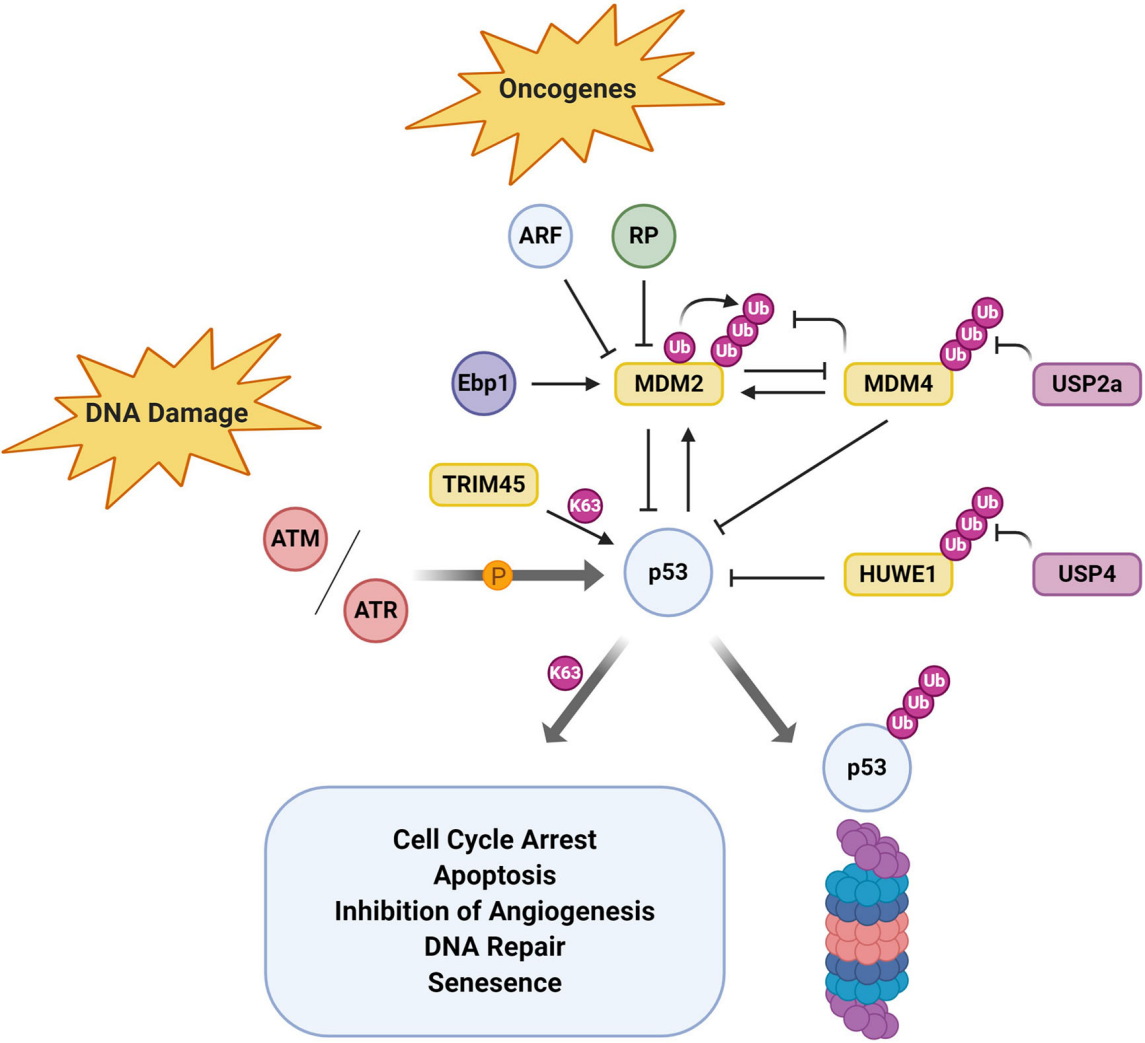
p53 Regulation by the UPS in Glioblastoma. Tumor suppressor p53 is subject to a plethora of upstream regulatory mechanisms including post-translational modification by ubiquitin. Here, E3 ubiquitin ligases and deubiquitinates that have been shown to modulate p53 function/activity in glioblastoma specifically are depicted.

The master-regulator p53 integrates various signaling pathways, relaying its tumor suppressive functions through a plethora of target genes. p53 is modified by a large variety of post-translational modifications which regulate its spatial and temporal expression. Ubiquitination of p53 was first discovered in the context of human papillomavirus, which highjacks the HECT E3 ligase E6AP to redirect its E3 ligase activity toward p53, an otherwise non-canonical substrate (92). In addition to oncogenic viruses, p53 is also targeted for ubiquitin-dependent degradation in multiple cancers. In GBM for example (**Figure 4**), p53 levels are regulated by the RING E3 ligase MDM2 as part of the ARF-MDM2-p53 axis which is dysfunctional in 84% of cases/94% of cell lines (294). Under normal physiological conditions, MDM2-p53 forms a negative feedback loop where p53 activation induces the expression of MDM2 which in turn promotes the ubiquitin-mediated degradation of p53 (295). This equilibrium is disrupted by *MDM2* amplification which negates p53 tumor suppressor function such as growth/cell cycle arrest, apoptosis or DNA repair. MDM4 performs a complementary role but lacks intrinsic E3 ligase activity (296). *Via* protein-protein interactions, MDM4 directly inhibits p53 by binding to its transcription activation and DNA binding domain (297, 298). In contrast to MDM2, MDM4 does not form homodimers but preferentially hetero-oligomerises with MDM2 *via* their C-terminal RING domains to mediate p53 ubiquitination (299). Indeed, heterodimer formation facilitates increased p53 ubiquitination but also stabilizes MDM2 by reducing its autoubiquitination. In GBM, homozygous deletions of *CDKN2A* (ARF/56%), gene amplification of *MDM2/4* (8.2%/9.4%) and missense mutations in *TP53* (31.5%) all lead to loss of p53 tumor suppressive functions, either through reduced activity or through reduced levels (TCGA, PanCancer Atlas).

Another interesting regulator of p53 activity in GBM is the DUB USP4 which negatively regulates p53 indirectly by stabilizing the HECT E3 ligase HUWE1/Mule (300). Although USP4 mRNA and protein levels were upregulated in GBM, its transient depletion did not result in changes in cell viability (252). In contrast, cells treated with the chemotherapeutic TMZ were significantly more sensitive to USP4 depletion by siRNA and showed decreased cell viability. This study suggests that USP4 mediates chemoresistance in GBM by preferentially inhibiting p53-mediated apoptosis. However, other E3 ligases such as TRIM45, may also positively regulate p53 activity. The TRIM family of RING E3 ligases are highly expressed in the brain, but TRIM45 mRNA and protein levels have been shown to be significantly downregulated in GBM tissue samples. TRIM45 had been previously shown to negatively regulate the MAPK and NFκB pathway, but its role as tumor suppressor had not been explored in GBM (301, 302). However, in GBM, TRIM45 mediated its tumor suppressor function through direct ubiquitination and stabilization of p53 (226). The authors suggested that K63-ubiquitination of p53 by TRIM45 inhibited subsequent degradative ubiquitination by for example MDM2/4.

### Stem Cell Maintenance

The discovery of cells with extensive proliferative and self-renewal capacity in AML gave rise to the cancer stem cell hypothesis (303). Glioblastoma stem cells (here defined as CD133^+^Nestin^+^) have since been identified as a distinct subpopulation, critical to tumorigenesis (304). The origin of GSCs may be disputable, but it is evident that the GSC subpopulation is key to the maintenance of tumor growth and invasive capacity, while also providing means for treatment resistance and thus GBM recurrence (305–307). Generally, GSC transcriptomic signature correlates with bulk tumor molecular subtype (excluding neural subtype), thus reflecting clonal heterogeneity and plasticity (308). Indeed, intra tumoral GSC subtype plasticity may allow for adaptation to a particular tumor niche or serves as a survival mechanism in response to microenvironmental cues. Identification of the underlying molecular mechanisms that drive stemness is thus key for successful therapeutic development. Using serial xenotransplantation and DNA barcoding, GSCs have been shown to exhibit a remarkable neutral proliferative hierarchy (309). In this model, a small pool of slow-cycling stem-like cells ensured tumor proliferative capacity by giving rise to rapidly-cycling progenitor cells. Although this model highlights the evolutionary fitness advantage of GSCs over non-GSCs, it does not take into account GSC plasticity. Indeed, multi-lineage plasticity not only extends to molecular subtypes but also exists as a dynamic equilibrium between GSCs and differentiated cancer cells (310). Stemness regulation by the tumor microenvironment results in a bidirectional equilibrium between CSC and non-CSC compartments and therefore GSCs should be regarded as reversible, transient state at the apex of a stem cell hierarchy (311, 312). GSC plasticity itself is now emerging as a key therapeutic target to overcome recurrence and drug resistance.

The underlying molecular mechanisms that contribute to GSC maintenance and plasticity, including the role of ubiquitin signaling, are still being worked out. E3 ligases/DUBs regulate the stability of key mediators of neuronal differentiation, including c-Myc, a core transcriptional regulator of GSCs. c-Myc levels are tightly controlled in a context-dependent manner by several E3 ligases and DUBs. One study showed that USP13 and SCF^FBXL14^ act as an E3-DUB pair regulating c-Myc ubiquitination in GBM (207). USP13 was found preferentially expressed in GSCs while SCF^FBXL14^ was predominantly expressed in non-stem glioma cells, enabling preferential stabilization of c-Myc in GSCs. USP28 was previously shown to stabilize c-Myc in HeLa and U2OS cells by antagonizing SCF^FBW7α^-mediated degradation and a more recent study has now reported its overexpression in GBM (269, 313). It has also been demonstrated that high expression of TRIP13, which stabilized c-Myc by inhibiting FBW7 transcription, correlates with poor patient survival (203). TRIM3 is another E3 ligase that has been shown to suppress c-Myc levels in GBM (219). In Drosophila, TRIM3 is an important regulator of asymmetric cell division, but whether its tumor suppressive effects in GBM are mediated through direct interaction with c-Myc remains to be shown (314).

The gene master regulator REST (repressor element 1-silencing transcription factor) is aberrantly expressed in brain tumors, where it likely maintains stem/progenitor cells through repression of neuronal genes (200, 201, 315). Here, the multi-subunit E3 ligase complex SCF^β-TrCP^ targets REST for proteasomal degradation *via* a phospho-degron. Although not in GBM specifically, USP7 has been demonstrated to counterbalance REST ubiquitination by SCF^β-TrCP^, facilitating neuronal differentiation in neural stem/progenitor cells. SCF^β-TrCP^ is a particularly versatile E3 ligase which has been implicated in several pathways including cell cycle regulation, NFκB and Wnt signaling (316). Interestingly, another study reported nuclear mislocalization of SCF^β-TrCP^ in GBM which led to reduced degradation of its cytosolic targets such as phospho-β-catenin (202). This may lead to increased Wnt signaling which is also commonly observed in GBM (317). Nevertheless, how SCF^β-TrCP^ regulation of REST and its nuclear mislocalization can be unified remains to be understood.

Another DUB enriched in GBM stem cells is USP1, which stabilizes the DNA damage response and stem cell maintenance regulators ID1/2 and Chk1 (246, 247). Radioresistance in CD133^+^ cells is conferred by preferential activation of the DNA damage response pathway *via* phosphorylation of checkpoint proteins ATM, Rad17, Chk1 and Chk2. Loss-of-function experiments on USP1 indeed resulted in impaired GSC survival and radiosensitization (305). Furthermore, CD133^+^ GSCs drive constitutive activation of the DNA damage response through high levels of replication stress not exhibited by CD133^-^ cells (318). In proneural glioma cells, where the PDGFR gene is frequently amplified, increased PDGF signaling drove expression of members of the E2F transcription factor family (E2F1-3). This in turn promoted E2F interaction with the USP1 promoter and increased USP1 levels which then stabilized the transcriptional regulator ID2 and maintained GSCs stemness (246, 247).

## The Ubiquitin-Proteasome System as a Source of Novel Therapeutics in GBM

Drug discovery has largely focused on developing enzyme inhibitors, in particular small molecular kinase inhibitors, with some success (319). Phosphorylation, like ubiquitination, is a reversible post-translational modification and high-throughput screens using small molecule libraries have identified vast numbers of kinase inhibitors that target either the catalytic ATP-binding pocket or adjacent hydrophobic cavities inhibiting substrate phosphorylation (320). The UPS has been dubbed as a new source of therapeutics although the development of small molecules inhibitors has been accompanied by inherent difficulties explaining the slow progress to date (30). E3 ubiquitin ligases may outnumber protein kinases but are inherently more difficult to target. Indeed, components of the UPS are exclusively found intracellularly which in comparison to the extracellular domains of receptor kinases, for example, negates antibody-based approaches. Moreover, E2-E3-substrate interactions are of transient nature and largely independent of well-defined binding pockets making high-throughput screens not readily applicable. Hence, the interface of E2-E3 interaction also does not lend itself to targeting. The identification as well as the fate and function of substrates modified by ubiquitin along with the mechanisms regulating E1, E2, E3 and DUB activity are still being defined. As we learn more about the specificity of enzymes in terms of the ubiquitin-dependent mechanisms they mediate and cellular processes they regulate, the ubiquitin system will offer a diverse therapeutic toolbox. This will be particularly important in the context of complex and heterogeneous pathologies such as GBM, where one “therapeutic magic bullet” might be difficult to achieve. Below we will summarize exciting developments targeting the components of the ubiquitin system and discuss the relevance of these strategies for GBM.

### Modulating Proteasomal Activity

Proteasome inhibition marked some of the earliest efforts in targeting the UPS. Since transformed cells exhibit higher proliferative capacity, ability to evade apoptosis and other regulatory mechanisms, these cells were more susceptible to proteasomal inhibition (321, 322). Proteasome inhibitors can be chemically divided into the general categories of peptide aldehydes, peptide vinyl sulfones, peptide epoxyketones, peptide boronates and lactacystin and its derivatives. However, only peptide boronates and epoxyketones bear the appropriate balance of potency, selectivity and metabolic stability required for clinical development (323). Nonetheless, proteasome inhibitors not suitable for the clinic have provided an invaluable understanding of cellular consequences of proteasomal inhibition, with the most prominent example being MG132 (carbobenzoxy-Leu-Leu-leucinal) (324).

Bortezomib (PS-341, Velcade), Carfilzomib (PR-171, Kyprolis) and Ninlaro (Ixazomib, Takeda) are currently the only FDA-approved proteasome inhibitors. All proteasome inhibitors share a similar mechanism of action, they bind active site threonine residues of the proteolytic β-subunits (325, 326). These structural studies identified the hydroxyl group of Thr^1^ as the catalytic nucleophile, which was confirmed by further crystal structures that demonstrated that only alanine but not serine substitution led to catalytic inactivity (327). The dipeptidyl boronic acid bortezomib (pyrazylcarbonyl-Phe-Leu-boronate) selectively targets the chymotrypsin-like activity of the proteasome with the boron atom forming a tetrahedral adduct with Thr^1^, exhibiting high potency (EC_50_ 0.6 nM) and a clinical-relevant cytotoxic profile (328). Treatment of various cancer cell lines resulted in cell cycle arrest in G_2_-M phase and subsequent apoptosis as evidenced by accumulation of cell cycle regulators p21 and p53 as well as other pro-apoptotic proteins (329). Even though the underlying mechanism remains to be fully elucidated, bortezomib is considered to inhibit NFκB activation by blocking the degradation of IκB and also increased sensitivity to chemotherapeutic agents (330). Even though a first phase I clinical trial in solid tumors yielded little success, a second phase I trial for hematologic malignancies showed promising results, ultimately leading to FDA approval of bortezomib in 2003 for multiple myeloma (331, 332).

In contrast to intravenous administration required for both bortezomib and carfilzomib, ixazomib (ninlaro) has become the first FDA-approved oral proteasome inhibitor (333). Ixazomib citrate is metabolized into active ixazomib which selectively and reversibly inhibits the chymotrypsin-like activity of the β5 subunit of the 20S proteasome. Nonetheless, drug delivery, in particular the ability to cross the blood-brain barrier, remains an issue for using proteasome inhibitors to treat brain pathologies such as GBM. Bortezomib was effective in GBM mouse models, but only when administered intracranially but not systemically (334). Proteasome inhibitors do not distinguish between normal and transformed cells which may result in non-specific cytotoxicity. Rather, they rely on the higher proliferative capacity of cancer cells to be more effective in this particular cell pool. However, new delivery strategies such as nanoparticle-derived systems may help overcome specificity issues by directing drugs to specific cellular compartments (335). Indeed, preclinical studies have highlighted the effectiveness and potential of bortezomib nanoparticle delivery, with for example anti-CD38 chitosan nanoparticles improving multiple myeloma cell targeting and resulting in a lower toxicity profile (336–338).

### Therapeutic Targeting of E1-Activating and E2-Conjugating Enzymes

Enzymes of the ubiquitination cascade also pose as promising targets for drug discovery. However, given there are only two main mammalian E1-activating enzymes (UBA1 and UBA6), inhibiting their function would also affect ubiquitin-dependent mechanisms as a whole. E1 enzymes carry out the ATP-dependent activation step resulting in the formation of a thiol ester bond between the ubiquitin adenylate and the active site cysteine residue (339). UBA1’s Cys^632^ has been successfully targeted *via* covalent modification by pyrazolidine-based inhibitors such as PYR-41 and PYZD-4409 (340, 341). Even though both showed selectivity for malignant cells, with the latter displaying potential for the treatment of hematologic malignancies, its mechanism of action and pharmacological properties are incompletely understood. Currently, the most promising candidate in development is MLN4924 (Pevonedistat) which is being evaluated in several phase I/II/III clinical trials (342). MLN4924 targets NEDD8 Activating Enzyme (NAE), which function as the initiator for the conjugation of ubiquitin-like modifier NEDD8. The small molecule inhibitor induces apoptosis due to accumulation of tumor-suppressive Cullin-RING ligase substrates and S-phase DNA synthesis dysregulation. Structural evidence suggests that MLN4924 inhibits NAE enzymatic activity by forming a NEDD8 adduct *via* its sulfamate moiety resulting in a NEDD8-AMP mimetic that occupies the adenylation active site (343).

In contrast to targeting the proteasome or E1-activating enzymes, other classes of enzymes in the ubiquitin system are likely to offer more specificity and therefore pose as more desirable therapeutic targets. With 40 E2 conjugating enzymes encoded in the human genome, this class of enzymes play an important role with regards to substrate specificity, in particular for RING E3 ligase-mediated ubiquitination. For example, the small molecule inhibitor CC0651 was originally identified in a screen for SCF^Skp2^, and exhibited dose-dependent inhibition of CDK inhibitor p27 (344). However, functional studies in budding yeast later revealed that the compound targets human UB2R1 (Cdc34) instead. Structural analysis further confirmed this and also identified CC0651 as an allosteric inhibitor, binding UB2R1 *via* its biphenyl ring system in a hydrophobic pocket distinct from the active site. Inhibition disrupts ubiquitin chain elongation but also stimulates autoubiquitination.

Another example is the E2 heterodimer UBE2N-UBE2V1 which has been successfully targeted by NSC697923 and BAY 11-7082 (345, 346). NSC697923 blocks ubiquitin transthioesterification by binding the active site cysteine residue of UBE2N (Ubc13), downregulating constitutive NFκB signaling in primary diffuse large B-cell lymphoma cells. BAY 11-7082 was thought to inhibit IκBα phosphorylation but here shown to inhibit K63 polyubiquitin chain formation by forming a covalent adduct with the UBE2N Cys^cat^. The compound exerts anti-inflammatory effects in primary B cell lymphoma and leukemic cells but is yet to undergo further preclinical evaluation.

### Therapeutic Targeting of E3 Ubiquitin Ligases

E3 ubiquitin ligases are at the pinnacle of the ubiquitination cascade, carrying out the final step. This makes them attractive drug targets due to their high degree of specificity and selectivity toward substrates. Nonetheless, the transient and dynamic nature of E3-substrate interaction and their lack of well-defined catalytic cavities makes them inherently difficult to target, especially with small molecule inhibitors. However, GDC-199 (venetoclax) which gained FDA approval for chronic lymphocytic leukemia (CLL) and small lymphocytic lymphoma (SLL) in May 2019 revived interest in the possibility of disrupting protein-protein interactions (347). Venetoclax selectively binds to BCL-2’s BH3-only protein hydrophobic binding groove, leaving the pro-apoptotic protein free to interact with for example BAX and BAK proteins, inducing mitochondrial membrane permeabilization and subsequent cell death (348, 349).

The F-box protein SKP2 is the substrate recognition subunit of the SCF^SKP2^ E3 ligase complex. Its well-defined role in several human malignancies, as well as availability of structural data, makes it a prime target for high-throughput screens. Indeed, *in silico* screens identified several hits which selectively target the p27 binding interface, while another screen identified compound 25 which disrupts SKP1 binding (350, 351). The compounds displayed significant effects on cell proliferation through various mechanisms including cell cycle arrest and suppression of Akt-mediated glycolysis in line with their respective targets.

The p53 regulator MDM2 is another well-studied drug target and particularly relevant in GBM where it is frequently amplified. MDM2 binds the transcriptional activation domain of p53 forming an autoregulatory feedback loop, which is complemented by direct binding of MDM2’s hydrophobic cleft to the amphipathic α-helix of p53’s transactivation domain (TAD) resulting in ubiquitination and subsequent proteasomal degradation (352, 353). A screen identified Nutlins, imidazoline analog, as potent and selective inhibitors of TAD binding, inducing p53 stabilization and downstream cell cycle arrest/growth inhibition (354). Crystal structures of imidazoline inhibitors in complex with MDM2 confirmed occupation of the TAD binding cleft as underpinning mechanism. Subsequently, further compounds disrupting MDM2-p53 interaction were identified. However, they suffer from the caveat that canonical ubiquitination of p53 mutants is MDM2-independent (355). An interesting example is AMG-232 (KRT-232) which averaged IC_50_s in the low nanomolar range in GBM cell lines and patient-derived GBM stem cells (356). More importantly, AMG-232’s suppressive effects seemed to extend selectively to GBM stem cells as the compound displayed efficacious inhibition of stemness-related factors Nestin and ZEB1 in a spheroid culture model. AMG-232 is currently being evaluated in 3 phase I clinical trials, with NCT03107780 probing its ability to penetrate GBM in patients with newly diagnosed or recurrent GBM. Several other imidazoline-based compounds are also currently undergoing early phase clinical trials with however so far modest clinical success (357).

Another class of compounds with several examples currently undergoing phase I clinical trials are inhibitors of apoptosis (IAP) antagonists (358). Proteins of the IAP E3 ligase family are endogenous inhibitors of apoptosis that sequester pro-apoptotic proteins such as caspases *via* their baculovirus IAP repeat (BIR) domain rendering them inactive (359, 360). Under physiological conditions, activation of the intrinsic apoptotic pathway induces Smac/DIABLO relocalization from the mitochondria to the cytosol where their binding to the hydrophobic BIR domain interface results in IAP dissociation and thus activation of pro-apoptotic proteins (361, 362). Efforts therefore focussed on generating Smac-mimetics, which bind the IAP BIR domain *via* the characteristic Ala–Val–Pro–Ile interaction motif (363). Smac-mimetics induce dimerization of IAP RING domains, an active conformation, resulting in autoubiquitination and subsequent degradation (364).

**Figure 5:**
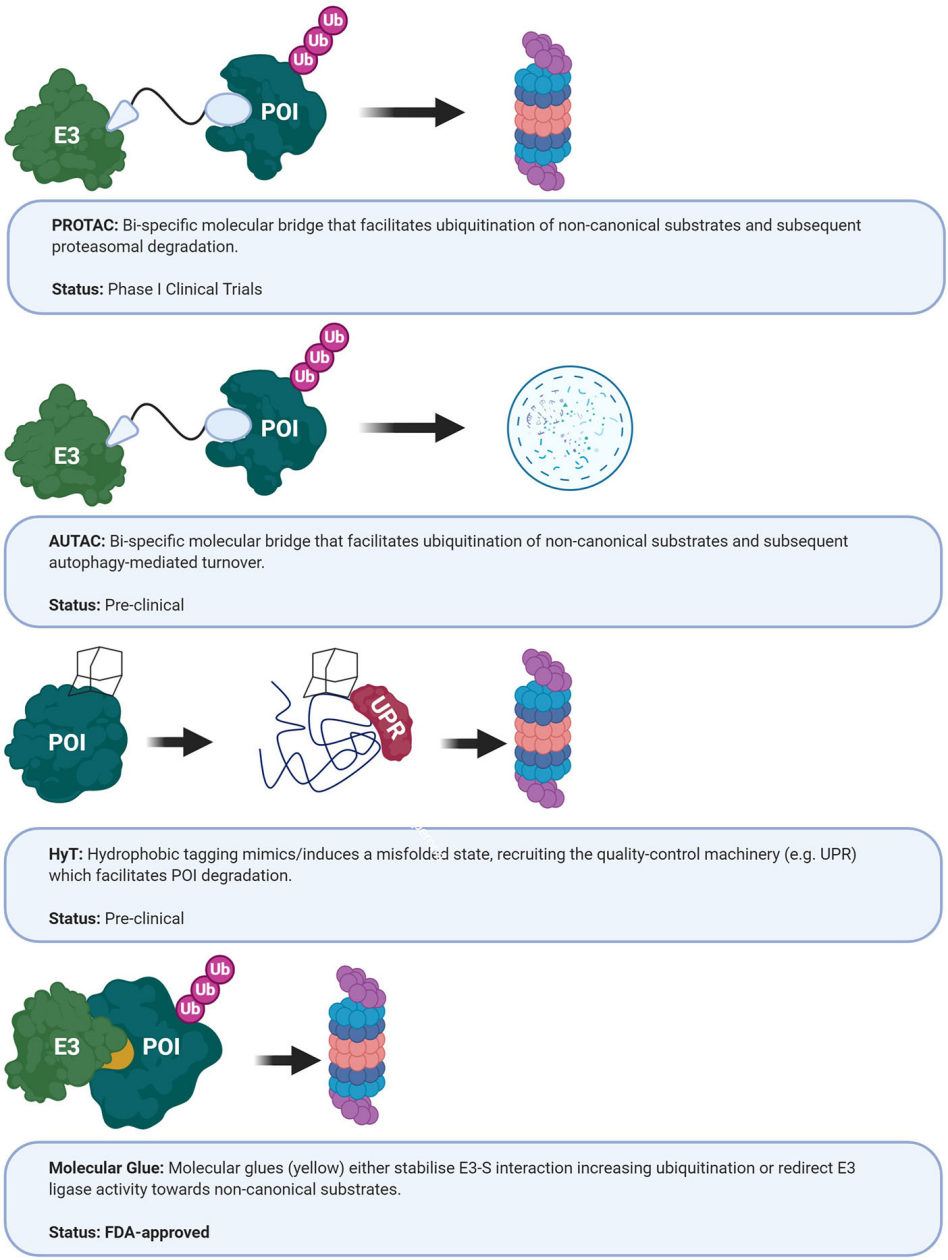
Current approaches targeting E3 ubiquitin ligases. AUTAC, autophagy- targeting chimeric molecules; HyT, hydrophobic tagging; POI, protein of interest; PROTAC, protein-targeting chimeric molecules; UPR, unfolded protein response (365–368).

The ability of small molecules to alter instead of inhibiting E3 ligase function has been demonstrated in nature as well as experimentally. For example, the plant hormones auxin and jasmonate function as so-called “molecular switches/glues” enhancing E3 ligase substrate affinity (**Figure 5**) (369, 370). The former is bound by the auxin receptor TIR1, an F-box component of the SCF multi-subunit complex, enhancing degradation of the downstream transcriptional regulators AUX/IAA. In addition, the thalidomide derivative lenalidomide was shown to alter the substrate specificity of Cereblon (CRBN) ubiquitin ligase, inducing the degradation of Ikaros family zinc finger proteins 1 and 3, B cell transcription factors, in multiple B cell malignancies (371, 372). These studies not only elucidate an important mechanism of these immunomodulatory drugs but also provide evidence that small molecules hold the potential to repurpose E3 ubiquitin ligases for targeting “undruggable” targets, in particular GBM-relevant oncoproteins such as c-Myc, β-catenin or MCL1 (373). Database mining and rational screening have been used successfully to identify molecular glue degraders that specifically target cyclin K, and these approaches will have broad applications for drug discovery (374, 375).

### Therapeutic Targeting of Deubiquitinases

With the previously outlined success of targeting the ubiquitination cascade, it is perhaps not surprising that deubiquitination is also an integral part of current drug discovery efforts. As previously discussed, DUB involvement is frequently observed in various cancers including GBM (**Table 2**). In an attempt to build on the success of bortezomib but also improve on issues associated with specificity, DUBs associated with ubiquitin hydrolysis at the 26S proteasome are currently evaluated. For example, the 19S subunit-associated DUB USP14, which is involved in ubiquitin recycling, is overexpressed in several diseases such as lung adenocarcinoma and non-small cell lung cancer (376, 377). In this context, the upregulation of USP14 is thought to maintain proteostasis of malignant cells through efficient protein degradation. The growth factor signaling transducer Akt phosphorylates USP14 on Ser^432^ resulting in an active conformation, thus providing means of globally regulating protein turnover (378). Similarly, UCHL5 is also associated with the 19S cap proteasome complex by binding to ubiquitin receptor RPN13 and functions by editing polyubiquitin degradation signals, cleaving distal ubiquitin moieties (379, 380). However, like USP14, UCHL5 is highly selective, promoting the degradation of certain proteins while guaranteeing the survival of others. For example, it was demonstrated that the RPN13-UCHL5 complex promotes degradation of inducible nitric oxide synthase (iNOS), while stabilizing NFκB suppressor IκBα (381). VLX1570 is a functional analog of the chalcone derivative b-AP15 with a piperidine to azepane ring substitution (382). b-AP15 was previously identified in a screen for lysosomal apoptosis pathway activation and displayed promising *in vivo* anti-tumor progression activity in several solid tumor models (383). Polyubiquitinated substrate accumulation led to USP14 and UCHL5 target identification and the compound being dubbed second-generation proteasome inhibitor. VLX1570 entered clinical trials in 2015 as a combination study with dexamethasone in myeloma patients, but despite continuous promising preclinical data, the trial had to be suspended in 2017 due to dose-limiting toxicity (NCT02372240). It will be interesting to see how other proteasomal DUB inhibitors fare, with several currently in preclinical development (384).

USP7 is another promising target for the treatment of various cancers as it regulates the stability of a multitude of oncoproteins and tumor suppressors (385). Many of which are also relevant in GBM and add to its previously discussed role in counterbalancing REST ubiquitination by SCF^β-TrCP^, facilitating neuronal differentiation in neural stem/progenitor cells (255). These include, for example, stabilization of FOXO (Forkhead box O) transcription factors, regulation of tumor suppressor PTEN nuclear-cytoplasmic partitioning or the p53 pathway (386–388). Several hits are currently investigated but share issues of selectivity and potency. One such compound, amidotetrahydroacridine derivative HBX 19,818, was shown to covalently bind active site Cys^223^ with an IC_50_ in the micromolar range (389). Experiments in cancer cell lines confirmed that similar to USP7 knockdown, HBX 19,818 promoted apoptosis and G1 phase cell cycle arrest as well as p53 stabilization. Similarly, P22077, previously identified during an activity-based proteomics screen, showed selective USP7 inhibition in an orthotopic neuroblastoma mouse model (390, 391). Xenograft growth was significantly inhibited *via* the USP7-MDM2-p53 axis. Recent structures of USP7 in complex with small molecule inhibitors should accelerate informed drug design and development. Importantly, it should be noted that like the previously described Nutlins, USP7 inhibitors are rendered ineffective when faced with p53 mutant malignancies which is the case for ≈32% of GBMs (TCGA, PanCancer Atlas).

USP15 has been implicated in NFκB, Wnt and TGF-β signaling, which are all recognized cancer pathways (392, 393). Building on a previous study that identified USP15 as DUB of receptor-activated SMADs, Eichhorn et al. established USP15 as SMURF2 counterpart (239, 394). USP15 gene amplification is commonly observed in GBM and correlates with aberrant TGF-β signaling. Currently, only weak USP15 inhibitors have been identified, but recent structural insights in its catalytic domain have provided a starting point for a more targeted approach (395). Similarly, USP1 is emerging as a candidate target in GBM, due to its increased expression in GSCs where it contributes to the DNA damage response and stem cell maintenance (247). The FDA-approved antipsychotic pimozide has been identified as USP1 inhibitor and is now being re-evaluated in various preclinical studies for cancer therapy (396). Also, the diphenylbutylpiperidine has CNS activity and was shown to induce radiosensitivity as well as chemosensitivity to TMZ treatment (397).

## Future Opportunities for GBM Therapeutics

In addition to small molecule inhibitors, several novel therapeutic avenues that exploit endogenous turnover machinery are being developed (**Figure 5**). Rather than delineating individual E2/E3-substrate pairings and subsequently subjecting the specific binding interface to a small molecule library screen, these new strategies co-opt endogenous protein degradation machinery – specifically the UPS (i.e. PROTACs, HyT, molecular glues), autophagy (AUTACs) and the endosomal/lysosomal (LYTACs) pathways (398). PROTACs are heterobifunctional molecules which can recruit E3 ubiquitin ligases to the desired protein targets, thereby co-opting the endogenous UPS for targeted protein degradation. Below, we will summarize how PROTACs work and also how some of these strategies provide new opportunities for GBM therapeutics.

### Protein-Targeting Chimeric Molecules

**Figure 6:**
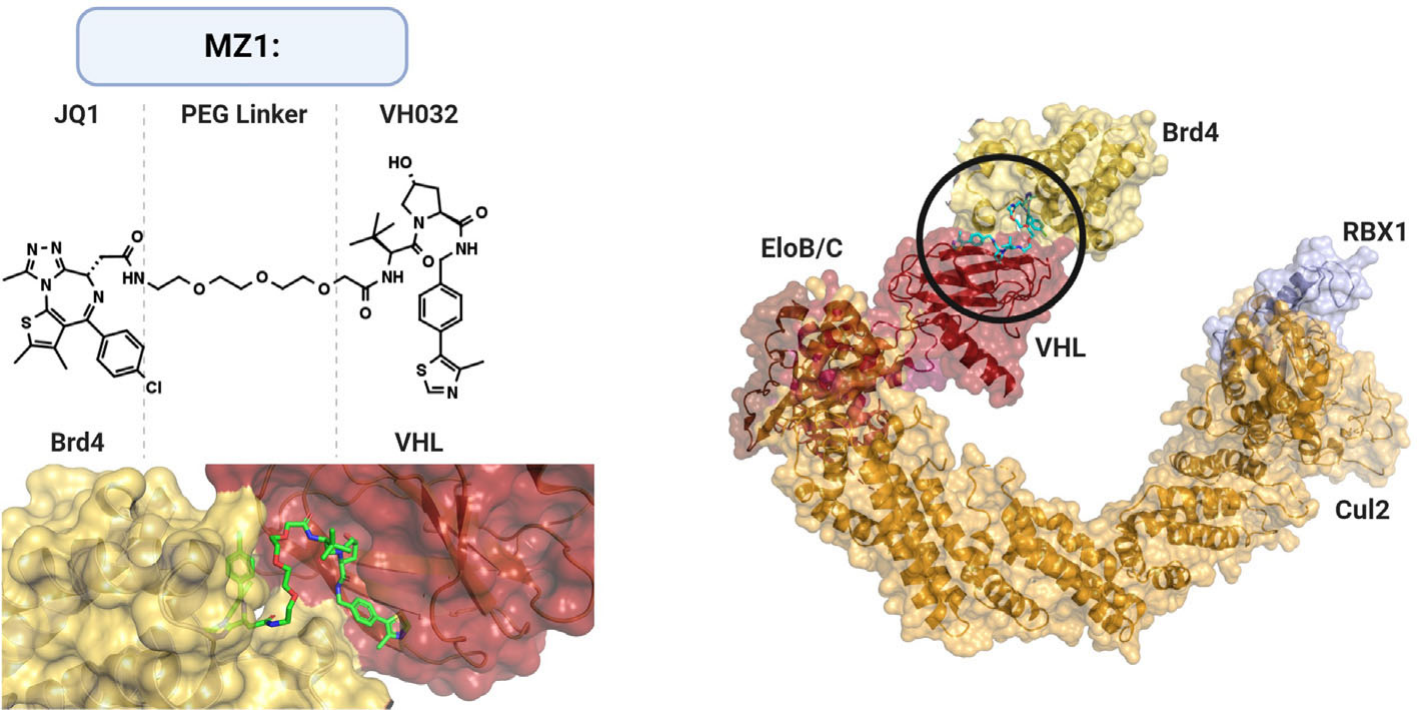
Structural Basis of PROTACs. PROtein-TArgeting Chimeric molecules (PROTACs) are heterobifunctional bridges that link E3 ligase activity to non-canonical substrates. PROTAC MZ1 links bromodomain inhibitor JQ1 to VHL ligand VH032 *via* a polyethylene glycol (PEG) linker. MZ1 facilitates binding and subsequent ubiquitination of BET (bromodomain and extraterminal) protein family member Brd4 (shown BRD4^BD2^) to cullin-RING ligase complex CRL2^VHL^. PDB: 5N4W, Cul2-Rbx1-EloBC-VHL ubiquitin ligase complex; 5T35, PROTAC MZ1 in complex with Brd4^BD2^ and pVHL : ElonginC:ElonginB.

PROTACs are an exciting new development in the field. They are bi-specific, artificial molecular bridges that facilitate ubiquitination of non-canonical substrates (**Figures 5**, **6**). Proof-of-concept was demonstrated in 2001, by using the artificial PROTAC-1 to target methionine aminopeptidase-2 (MetAP-2) to SCF^β-TrCP^ for proteasomal degradation in *Xenopus laevis* egg extracts (365). β-TrCP is the substrate recognition domain of SCF^β-TrCP^, endogenously recognizing a short, phosphorylated peptide stretch within IκBα resulting in subsequent ubiquitination, degradation and thus activation of NFκB signaling (399). MetAP-2 is a primary target of ovalicin (OVA), which covalently binds the active site His^231^ resulting in a downstream inhibitory effect on endothelial cell proliferation (400). The PROTAC-1 design combines both moieties, the IκBα phosphopeptide and OVA, resulting in the molecular bridging of MetAP-2 and SCF^β-TrCP^, two otherwise functionally unrelated proteins. Subsequently, Sakamoto and colleagues designed two similar PROTACs using estradiol and dihydroxytestosterone (DHT) instead of OVA, targeting estrogen receptor (ER) and androgen receptor (AR), respectively (401). The experiments carried out in HEK-293 cells provided important *in vitro* validation. Issues with poor cell permeability were overcome with PROTAC-4 which was developed by ARIAD Pharmaceuticals. It included a poly-D-arginine tag (-ALAPYIP-(D-Arg)_8_-NH_2_) facilitating improved cell permeability and eliminating the previous need for microinjection (402). Nonetheless, complex synthetic chemistry and low efficacy were issues that remained.

However, *in vivo* application and thus revival of the technology became achievable with the development of E3 ligase-specific ligands. These second-generation PROTACs relied on small molecules rather than peptides for E3 ligase recruitment. The first examples included MDM2, cIAP1 and Cullin-Ring ligase (CRL) complex substrate receptors such as CRBN and VHL (403–409). The Crews group who, along with the Deshaies group, first reported PROTAC in 2001, developed its second-generation AR-targeting PROTAC by coupling Nutlin to a selective androgen receptor modulator (SARM) *via* a polyethylene glycol (PEG) linker (407). Nutlin targets the AR for proteasomal degradation by binding to the p53 interaction interface of MDM2 (354). In 2014, thalidomide and its derivatives lenalidomide and pomalidomide were shown in complex with the E3 ligase DDB1–CRBN, thus validating the E3 ligase as the target of the immunomodulatory drugs (410, 411). The phthalimide ring system found in thalidomide and its derivatives was also utilized for DDB1–CRBN recruitment in a PROTAC design for the degradation of bromodomain and extra-terminal (BET) proteins (403). The other half of the PROTAC consisted of the competitive bromodomain inhibitor JQ1 which binds in the acetyl-lysine binding cavity of BRD4 (**Figure 6**) (412). The hybrid molecule termed dBET1 displayed *in vivo* efficacy in a human leukemia xenograft model and induced a more robust apoptotic response in primary human leukemic blast cells compared to BRD inhibition. Similarly, the improved pharmacodynamics could be replicated by another BRD4-targeting PROTAC, ARV-825, in Burkitt’s lymphoma cell lines showing promising results for MYC-driven malignancies (404).

Conceptually similar to PROTAC, hydrophobic tagging (HyT) utilizes a hydrophobic moiety instead of a peptide/small molecule as E3 ligase recruiting domain (**Figure 5**) (366). Since functional proteins fold in a manner that conceals hydrophobic side chains to assume a lower energy state, the additional hydrophobic surface group is thought to mimic/induce a misfolded state leading to proteasomal degradation (413, 414). The mechanism is not fully elucidated yet, but it is thought that HyT modification initially recruits the chaperone machinery in an attempt to refold the protein, although ultimately targeting HyT-modified proteins to the proteasome (415). Feasibility of the approach was demonstrated by the addition of the cycloalkane adamantane to a bacterial dehalogenase (HaloTag) which resulted in robust degradation of HaloTag fusion proteins in culture and mice (366). Similarly, the pseudo-kinase Her3 which is considered “undruggable” by ATP-competitive small molecules was found to be targetable for degradation by derivatization of the selective ligand TX1-85-1 with the hydrophobic adamantyl moiety (416).

However, many questions remain with regards to clinical application. For example, the large molecular weight of PROTACs may pose challenges to oral bioavailability, pharmacokinetics and tissue specificity. Nonetheless, preclinical evidence are convincing, particularly for the two recently developed BET family protein-targeting PROTACs which exhibit EC_50_s in the low picomolar range, QCA570 and compound 23 (417, 418). Furthermore, Sun et al. demonstrated that oral, as well as intraperitoneal PROTAC delivery, can mediate robust and global FKBP12 and Bruton’s tyrosine kinase (BTK) degradation in animals from mice to rhesus monkeys (419). Here, the PROTAC RC-32 rendered FKBP12 undetectable after only one day in most organs except the brain, indicating its inability to cross the blood-brain barrier. However, mice treated *via* intracerebroventricular (i.c.v.) injection displayed localized FKBP12 degradation in the brain, potentially expanding the use of PROTACs to GBM as well as other brain diseases including neurodegenerative disorders such as Alzheimer’s disease. It will be interesting to see how the PROTACs fare in the first clinical trials. The AR-targeting ARV-110 and ER-targeting ARV-471 (Arvinas) are currently undergoing recruitment for phase I clinical trials against prostate and breast cancer, respectively (NCT03888612/NCT04072952). A recent update at the American Association for Clinical Oncology (ASCO) suggests that ARV-110 showed antitumor activity and reduced PSA levels in some patients (J Clin Oncol 38: 2020 (suppl; abstr 3500)).

## Conclusions

GBM remains the deadliest cancer with limited therapeutic options. Recent discoveries that are starting to define its heterogeneity indicate that similar to other cancers, personalized therapies will be the way forward. Protein degradation is a ubiquitous feature that is essential to maintain cellular homeostasis, and the small protein modifier ubiquitin plays a key role in regulating protein fate and function, and thereby impacts on most signal transduction pathways and cellular processes.

In this review we have summarized components of the ubiquitin system which are found deregulated in GBM as well as highlighted key molecular mechanisms involved. In just over twenty years or so since the first reports of the discovery of the UPS, PROTACs have shown some exciting potential by being able to control the fate of proteins and trigger their degradation on demand. This has stirred new hopes for effective targeting of the many oncogenic proteins that have been identified as drivers of disease, in particular, those that were dubbed “undruggable”. It has nevertheless taken almost another 20 years to bring PROTACs and targeted protein degradation to the forefront of drug discovery. Recent developments in chemical biology, synthetic biology as well as the first ongoing clinical trials will no doubt accelerate the delivery of new therapies.

The examples included in our review aim to showcase the diversity of ubiquitin-dependent molecular mechanisms that are now being targeted as well as the fast-expanding toolbox of ubiquitin-based therapeutics that are becoming available. PROTACs are prime examples and they are already being adapted to oncoproteins also relevant for GBM including BRD4 (Myc) (**Figure 6**), ERK1,2 (MAP kinase pathway), EGFR and CDK4/6 (404, 420–423). PROTACs have so far only been designed based on a small number of Cullin-RING E3 ligases, leaving a large number of E3 ligases implicated in GBM still available for investigation (**Table 1**). Indeed, the tissue-specific expression of E3 ligases used in PROTACs will need to be ascertained as well as the impact that drafting an endogenous E3 ligase for therapeutic help might have on the system. Further exciting developments include combining optogenetics with protein degrader strategies such as Opto-PROTACs, as this could provide added control over the timing and induction of protein degradation (424).

These technological advances will no doubt offer new avenues for GBM where little therapeutic progress has been made throughout the last decades. Ubiquitin-dependent mechanisms have been implicated in the regulation of most if not all hallmarks of GBM, in particular the signal transduction pathways that confer cancer cells properties but also stemness and heterogeneity which have so far hindered the use of potential treatments through mediating drug resistance. Results from the first PROTAC clinical trials are eagerly awaited to inform on pharmacological viability and to outline future hurdles in the field. In the context of GBM and other brain tumors, it will also be important to improve drug delivery systems that could overcome or bypass the blood-brain barrier such as nano-vehicles, strategies for enhancement of brain permeability, active transporter or alternative administration regimens [reviewed in (425, 426)].

## Author Contributions

NS and JL designed the content of the review with input from all the co-authors. NS wrote the review with feedback from all the co-authors. All authors contributed to the article and approved the submitted version.

## Funding

NS’s PhD studentship is funded by a GW4 BioMed MRC Doctoral Training Partnership. We also acknowledge funding from the University of Bath Alumni for a GBM pump-priming grant for some of the work carried out in the Licchesi laboratory.

## Conflict of Interest

The authors declare that the research was conducted in the absence of any commercial or financial relationships that could be construed as a potential conflict of interest.
